# Utilizing Nanomaterials in Microfluidic Devices for Disease Detection and Treatment

**DOI:** 10.3390/nano15060434

**Published:** 2025-03-12

**Authors:** Zhibiao Tian, Yatian Fu, Zhiyong Dang, Tao Guo, Wenjuan Li, Jing Zhang

**Affiliations:** 1 College of Basic Medicine, Hebei University, Baoding 071000, China; ttt_2820@163.com (Z.T.); dzy5080590@163.com (Z.D.); 2 Department of Biomedical Engineering, City University of Hong Kong, Hong Kong, China; yatianfu2-c@my.cityu.edu.hk; 3 Hong Kong Centre for Cerebro-Cardiovascular Health Engineering (COCHE), Hong Kong, China; 4 Key Laboratory of Pathogenesis Mechanism and Control of Inflammatory-Autoimmune Diseases in Hebei Province, Hebei University, Baoding 071000, China

**Keywords:** nanomaterials, microfluidic, rapid detection, disease models, treatment

## Abstract

Microfluidic technology has gained widespread application in the field of biomedical research due to its exceptional sensitivity and high specificity. Particularly when combined with nanomaterials, the synergy between the two has significantly advanced fields such as precision medicine, drug delivery, disease detection, and treatment. This article aims to provide an overview of the latest research achievements of microfluidic nanomaterials in disease detection and treatment. It delves into the applications of microfluidic nanomaterials in detecting blood parameters, cardiovascular disease markers, neurological disease markers, and tumor markers. Special emphasis is placed on their roles in disease treatment, including models such as blood vessels, the blood–brain barrier, lung chips, and tumors. The development of microfluidic nanomaterials in emerging medical technologies, particularly in skin interactive devices and medical imaging, is also introduced. Additionally, the challenges and future prospects of microfluidic nanomaterials in current clinical applications are discussed. In summary, microfluidic nanomaterials play an indispensable role in disease detection and treatment. With the continuous advancement of technology, their applications in the medical field will become even more profound and extensive.

## 1. Introduction

Microfluidic technology has a wide range of applications and serves as a pivotal tool in biochemical and medical domains, facilitating drug screening, cell culture, and disease diagnosis [1,2,3]. Its advantages over traditional experimental methods are strikingly apparent [4]. Microfluidic technology enables rapid sample processing and precise manipulation of liquids in experiments. There are various flow control operation modes in microfluidic technology. Pressure-driven flow refers to the movement of fluid within microchannels induced by the application of external pressure [5]. Electrokinetic flow primarily comprises electroosmosis and electrophoresis. Electroosmosis is the phenomenon where an external electric field is applied to a channel to form an electric double layer, thereby driving the fluid movement within the channel [6]. Electrophoresis refers to the migration of charged particles in the fluid due to an electric field [7]. Magnetic manipulation achieves control of liquids by introducing magnetic nanoparticles (NPs) under a magnetic field [8]. The functionality and accuracy of the chip are maintained through the meticulous design of channels, chambers, and other structures [9]. The advancement of microfluidic technology has led to the development of advanced bioanalysis systems for detecting disease-related biomarkers. With ongoing research progress, various organ chips have been created using microfluidic technology to replicate the physiological environment of the human body [10,11], including brain chips [12], vascular chips [13], and tumor chips [14]. This technology serves as a robust platform for in vitro drug screening and delivery. Nanomaterials can be easily incorporated into microfluidic channels as capture and signaling elements to enhance sensing or therapeutic capabilities. Notably, microfluidic cell culture models integrated with nanomaterials can substitute for in vivo disease models in nano-therapy screening. Nanomaterials have excellent surface effects and good biocompatibility and biodegradability [15,16,17,18], which make them widely used. Nanomaterials can serve as tools for disease biomarker detection and disease treatment [19,20,21]. In these fields, the application of nanomaterials achieves high sensitivity and high specificity of multifunctional detection [22] and reduced detection times [23]. Moreover, nanomaterials enhance drug bioavailability and targeting [24], thereby substantially amplifying therapeutic efficacy [25].

**Figure 1:**
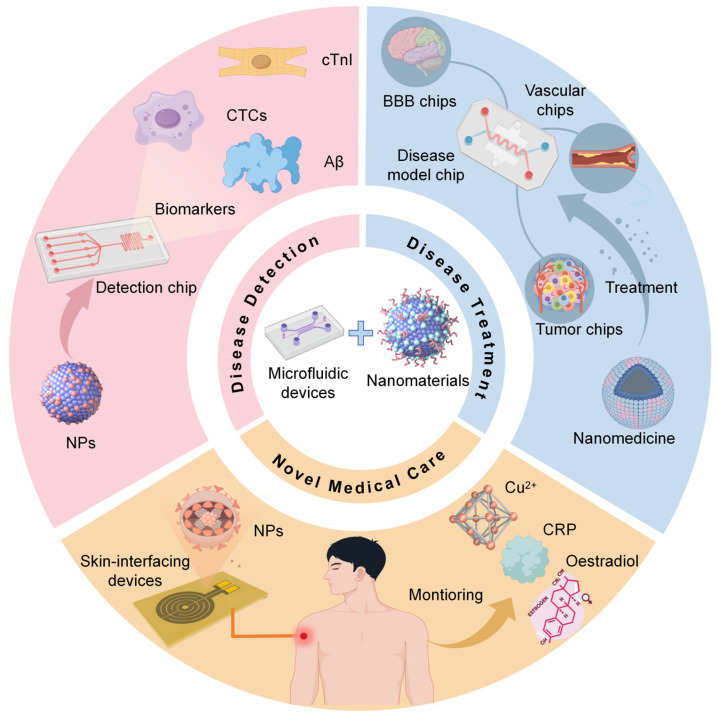
Integrating microfluidic devices and nanomaterials in disease detection, treatment, and novel medical care encompasses cardiovascular diseases, neurological disorders, and tumors. Detection of diseases entails identifying various biomarkers. Disease treatment involves utilizing microfluidic chips to develop different models for studying the effectiveness of nanomedicine. Novel medical care implements skin interface devices for monitoring a variety of indicators. (By Figdraw.).

Early diagnosis and prompt treatment of numerous diseases have demonstrated a profound impact on patients’ survival rates. For instance, in the case of cancer patients, timely diagnosis and appropriate treatment can lead to a 20% reduction in mortality [26]. Nevertheless, existing detection techniques like computed tomography (CT) necessitate large, specialized equipment, carry a high false positive rate, and entail radiation risks [27,28]. Simultaneously, liquid biopsy, while promising, imposes stringent demands and prolonged detection times for identifying biomarkers [28], posing a substantial hurdle in achieving rapid and accurate early detection and treatment. Cardiovascular diseases often rely on electrocardiograms [29], but the efficiency and accuracy of these methods are significantly lower than standard laboratory tests. The detection of neurological diseases also faces limitations with traditional imaging techniques [30], as well as a lack of technology and components for identifying specific biomarkers [31]. Given these challenges, the imperative to devise novel detection and treatment strategies is paramount. In recent times, the fusion of microfluidic technology with nanomaterials has emerged as a trend in biomedical research. This integration of microfluidic devices and nanomaterials has exhibited remarkable promise in disease diagnosis and treatment [32,33], offering a fresh perspective on addressing the challenges outlined above. The synergy of these two technologies can be categorized into two main facets. Firstly, by leveraging the unique properties of nanomaterials, including magnetic, optical, or electrochemical characteristics, along with their high surface area-to-volume ratio and easy functionalization, microfluidic devices can be enhanced [34,35]. Incorporating nanomaterials with distinct morphologies and multifunctional surface properties into microfluidic systems enables the rapid and precise detection of disease biomarkers [36]. Secondly, in the realm of in vitro research, organ chips fabricated through microfluidic devices technology have the capacity to replicate disease microenvironments. These chips serve as valuable platforms for investigating the therapeutic impacts of nanomedicines on diseases, enabling the assessment of both efficacy and safety [37]. Furthermore, organ chips play a pivotal role in nanomedicine research by facilitating the screening of nanomedicines, a critical step in the advancement of novel drug development [38]. This article provides a comprehensive overview and synthesis of the latest advancements in utilizing nanomaterials in combination with microfluidic devices for disease detection and treatment over the last five years (Figure 1). The article discusses the specific functions of nanomaterials and the advantages of their combination with microfluidic devices. Furthermore, the article demonstrates the integration of nanomaterials with microfluidic devices in innovative medical equipment, particularly devices for skin interaction and imaging technologies. Lastly, the article evaluates the challenges encountered in merging microfluidic devices and nanomaterials for clinical use and puts forth potential directions for future development.

## 2. Application of Microfluidic Technology Integrated with Nanomaterials in Disease Detection

The detection of biomarkers plays a crucial role in the early diagnosis of diseases, the evaluation of personalized treatments, and the assessment of prognosis [39,40,41]. By harnessing microfluidic devices integrated with nanomaterials (Table 1), a new pathway for disease biomarkers detection has been paved. This advanced technology not only significantly reduces the time required for detection but also greatly enhances the sensitivity of detection, thereby promoting advancements in the field of clinical diagnostics.

### 2.1. Detection of Cardiovascular Disease Biomarkers

Cardiovascular diseases (CVD), primarily including myocardial infarction, hypertension, and coronary artery disease, are among the leading causes of death and disability worldwide. The incidence and mortality rates of CVD have been increasing annually. According to the World Health Organization (WHO), cardiovascular diseases are responsible for approximately 17.9 million deaths each year, accounting for 32% of global mortality [62]. Risk factors for cardiovascular diseases include obesity, smoking, diabetes, and genetic predispositions [63]. Numerous studies in the past have identified biomarkers associated with cardiovascular diseases, and the detection of these biomarkers can assist in the early diagnosis of CVD [64,65]. Nanomaterial-enhanced microfluidic devices offer advantages such as rapid response and high sensitivity in detecting cardiovascular disease biomarkers [66,67]. This is mainly attributed to the high specific surface area, quantum size effect, and good biocompatibility of nanomaterials, which enable them to provide more active sites and enhance the strength of detection signals [68]. This offers more accurate and faster detection methods for clinical diagnostics.

**Figure 2:**
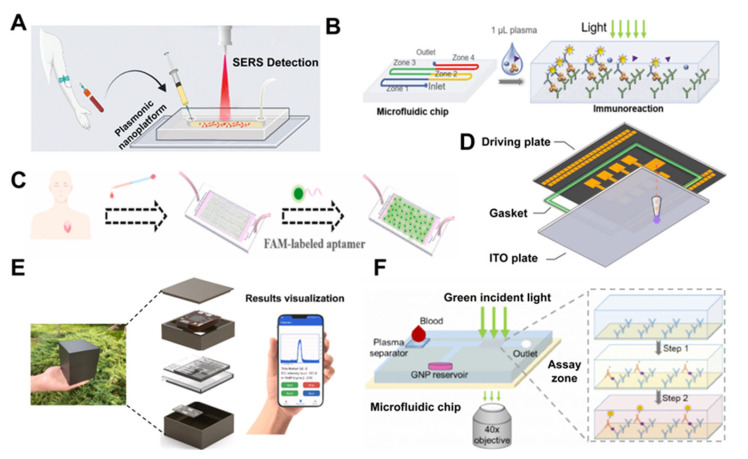
Microfluidic-based biomarkers detection using NPs in the cardiovascular system. (**A**) The schematic illustration of the biosensor designed for detecting the cTnI biomarker. (The image is reproduced from Campu et al. [32], licensed by CC BY.) (**B**) The multizone microfluidics chip utilizes gradient-based digital immunoassay for the detection of hs-cTnT. The light source is used to excite and detect gold NPs. (Reprinted with permission from reference [42]. Copyright 2021 American Chemical Society.) (**C**) Schematic diagram of herringbone microfluidic chip integrated with multifunctional barcodes for capture and detection of CVD biomarkers. (The image is reproduced from reference [43] with permission from Elsevier, copyright 2024.) (**D**) The DMF chip is compris4e of the top driving plate and the gasket bottom indium tin oxide plate. The side view illustrates that the droplet is loaded from the inlet hole and ejected by silicone oil. (The image is reproduced from reference [44] with permission from Elsevier, copyright 2024.) (**E**) The electrochemiluminescence device integrates a microfluidic chip and an embedded electronic system for point-of-care testing of acute myocardial infarction. (The image is reproduced from reference [45] with permission from Elsevier, copyright 2023.) (**F**) Schematic diagram of a microfluidic digital immunoassay for NT-proBNP instant detection. First, the assay zone is functionalized with a capture antibody. The blood mixed with biotinylated detection antibodies is transported to the assay zone, where immune complexes are formed. Then, streptavidin-conjugated GNPs are released from the reservoir, labeling the immune complexes, which are recorded through bright-field imaging. (Reprinted with permission from [46]. Copyright 2024 American Chemical Society.).

Cardiac troponin is a crucial biomarker for the detection of acute myocardial infarction [69]. Campu et al. developed a portable microfluidic plasmonic chip (Figure 2A) for the rapid and real-time detection of cardiac troponin I (cTnI) biomarkers [32]. They fabricated a plasmonic nanoplatform based on immobilized gold nanobipyramids, which possess both optical and thermal plasmonic properties, enabling strong light scattering and absorption, leading to Localized Surface Plasmon Resonance (LSPR). This resonance can enhance the local electromagnetic field around the NPs, thereby amplifying the Surface-Enhanced Raman Spectroscopy (SERS) signal. By employing a sandwich immunoassay approach, this platform was utilized as a substrate to capture cTnI, significantly facilitating its detection. Compared to traditional enzyme-linked immunosorbent assays (ELISA), this method demonstrated high sensitivity and specificity. Notably, the detection of cTnI using this approach requires only five minutes. This research has been validated in clinical applications, advancing the early diagnosis of heart disease. Regarding the sample size requirements for cardiac troponin detection, Jing et al. reported a gradient-based digital immunoassay method for detecting high-sensitivity cardiac troponin T (hs-cTnT), which requires only 1 μL of plasma sample to achieve highly sensitive detection of hs-cTnT [42]. The study developed a multi-region microfluidic channel for capturing C (Figure 2B). When hs-cTnT binds to the capture antibody, a concentration gradient of hs-cTnT forms along the channel due to the limited amount of hs-cTnT. Subsequently, a biotin-labeled detection antibody and streptavidin-coated gold NPs were used for the immunoassay, forming an immune complex. Finally, the concentration of cTnT in the sample was quantified by detecting and comparing the number of bound gold NPs in different regions. This gradient-based digital immunoassay method holds significant potential for clinical testing and may also be suitable for the detection of other biomarkers.

In addition, Li et al. proposed a herringbone microfluidic chip (Figure 2C) that integrates photonic crystal (PhC) barcodes with a core–shell structure, functionalized with oligonucleotides, for high-throughput multiplex detection of CVD biomarkers [43]. The study utilized carboxylated single-walled carbon nanotubes (CSWCNs) and silicon NPs to fabricate the PhC barcodes. The introduction of silicon NPs enhanced the mechanical strength and hydration of the photonic crystal barcodes, which helps maintain the stability of the encoded information in complex fluid environments while also providing carboxyl groups for probe immobilization, thereby improving the capture efficiency of biomarkers. The chip employs a herringbone microfluidic channel design to enhance fluid vortex resistance and contact frequency, which increases sample capture efficiency and detection sensitivity. The study selected three common clinical biomarkers (myoglobin Myo, cardiac troponin I cTnI, and B-type natriuretic peptide BNP), with detection limits of 0.0263 ng/mL, 0.0150 pg/mL, and 0.1432 ng/mL for Myo, BNP, and cTnI, respectively. Comparison with clinical results demonstrated the high efficiency, sensitivity, and specificity of the core–shell PhC barcode-integrated herringbone microfluidic system. The developed platform is currently limited to fluorescence imaging-based multiplex detection and should be extended to colorimetric methods integrated with smartphones in the future.

Heart-type fatty acid-binding protein (h-FABP) has emerged as a superior biomarker for acute myocardial infarction (AMI) compared to Myo and cTnI, owing to its tissue specificity and low molecular weight [70,71]. Chen et al. proposed a novel biosensor based on aggregation-induced emission nanoparticles (AIENPs) (Figure 2D) for the rapid and sensitive detection of h-FABP on a digital microfluidic workstation [44]. This biosensor employs a sandwich immunoassay, where AIENPs and magnetic NPs serve as fluorescent probes and capture particles, respectively, covalently bound to h-FABP-specific antibodies. Due to their unique aggregation-induced emission properties, AIENPs can specifically activate their fluorescence upon nanoparticle aggregation, thereby enhancing the sensitivity of h-FABP detection. The digital microfluidic workstation utilizes the electrowetting effect to precisely control microdroplets, enabling automated detection of h-FABP from serum samples, with the entire analytical process taking only 15 min.

Meanwhile, with the continuous advancement of detection technologies, to achieve on-site rapid detection of h-FABP, Zhu et al. developed an integrated microfluidic chip-based electrochemiluminescence (ECL) device (Figure 2E) [45]. The study employed Ru(bpy)_3_^2+^-loaded silica NPs as ECL probes. The microfluidic chip facilitated the mixing of samples with ECL probes and the subsequent immune reaction. An integrated electronic system provided voltage stimulation and photon detection, while a smartphone application enabled the visualization of test results. The detection limit for h-FABP was 0.72 ng/mL, and the accuracy of the device was validated using clinical human serum samples. The device can also be adapted for the detection of other biomarkers by altering the antibodies.

N-terminal pro B-type natriuretic peptide (NT-proBNP) is also an important biomarker for the clinical diagnosis of heart failure [72]. Chen et al. developed a point-of-care (POC) compatible microfluidic digital immunoassay (Figure 2F) capable of quantifying NT-proBNP concentrations in small volumes of whole blood [46]. They utilized streptavidin-coated gold nanoparticles (GNPs) to label surface-bound individual NT-proBNP immune complexes, and bright-field microscopy was employed to record the GNP-labeled immune complexes. The NT-proBNP concentration in the sample was quantified using a digital/analog calibration curve. The device could detect NT-proBNP in the range of 8.24–10,000 pg/mL from 7 μL of whole blood within 10 min, with a detection limit of 0.94 pg/mL. The feasibility of this method was validated through comparison with other detection methods, offering a novel POC approach for the clinical detection of heart failure.

When the body is infected, the concentrations of procalcitonin (PCT) and interleukin-6 (IL-6) increase to varying degrees, and the levels of these two biomarkers can serve as important indicators of infection. Wu et al. developed a highly sensitive immunoassay based on a microfluidic chip, which incorporates a streptavidin-biotin-eroxidase (SA-B-HRP) nanocomplex signal amplification system (MIS) for the simultaneous detection of PCT and IL-6 [47]. SA exhibits extremely high affinity for B, and with its four binding sites, SA can efficiently bind biotin-labeled enzymes, antibodies, and other molecules, allowing a large number of horseradish peroxidase (HRP) molecules to be immobilized at the reaction sites. After the binding reaction of PCT and IL-6 with the immobilized antibodies, signal amplification is achieved using biotin-labeled detection antibodies and the SA-B-HRP nanocomplex. The application of SA-B-HRP significantly enhances the sensitivity of traditional microfluidic immunoassays, advancing the clinical application of MIS.

By employing highly sensitive immunoassay methods combined with microfluidic technology utilizing nanomaterials, rapid detection of cardiovascular disease biomarkers such as cardiac troponin has been successfully achieved. These methods are characterized by their rapid response and high sensitivity, playing a crucial auxiliary role in the clinical rapid diagnosis of cardiovascular diseases. Future research will focus on further enhancing the sensitivity and convenience of detection, aiming to provide more efficient tools for the diagnosis and treatment of cardiovascular diseases.

### 2.2. Detection of Neurological Disease Biomarkers

Neurological disorders are a category of diseases that affect the brain, spinal cord, and peripheral nervous system, such as Alzheimer’s disease (AD), Parkinson’s disease (PD), and multiple sclerosis (MS). These disorders often lead to symptoms such as cognitive dysfunction, motor dysfunction, and sensory abnormalities, severely impacting patients’ quality of life [73,74,75,76]. Novel biomarkers for AD include amyloid β (Aβ), phosphorylated tau protein (P-tau), and glial fibrillary acidic protein (GFAP). The detection of these biomarkers has shown significant potential in both clinical and research settings [77,78].

**Figure 3:**
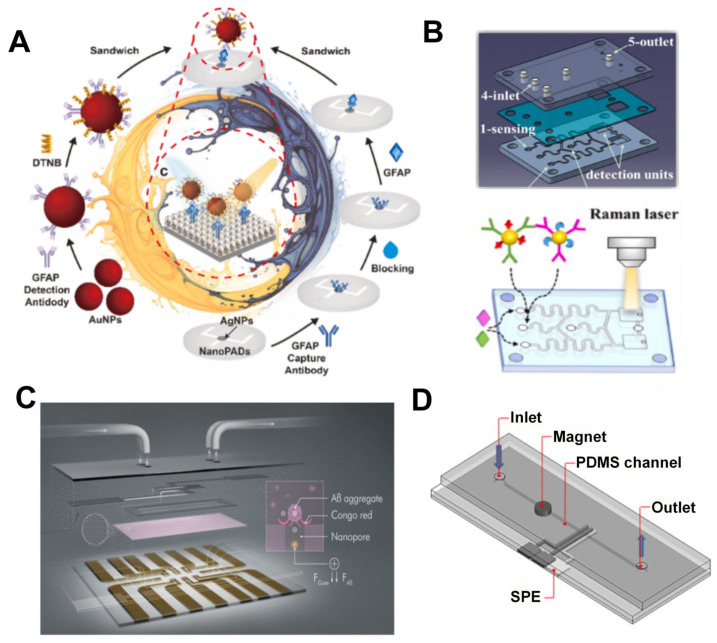
Enhanced detection in the nervous system through nanomaterials in conjunction with microfluidic devices. (**A**) Schematic illustration of the NanoPAD for SERS-based immunoassay targeting the detection of AD biomarker GFAP. The figure on both sides shows the fixation of GFAP capture antibodies on NanoPADs and the SERS detection process of the sandwich complex formed by the GFAP antigen and the detection antibodies labeled with DTNB-AuNPs. (The image is reproduced from reference [48] with permission from Elsevier, copyright 2024.) (**B**) Schematic diagram of PS/Au-based SERS immunosensor chip for sensitive detection of Aβ1-42 and Tau protein. (The image is reproduced from reference [49] with permission from Elsevier, copyright 2023.) (**C**) Schematic of the μf-OECT for Aβ detection. The figure highlights the nanoporous membrane functionalized with Congo red for capturing Aβ aggregates, which modulates the transistor’s characteristics upon binding, enabling sensitive detection within a wide concentration range in human serum samples. (The image is reproduced from Koklu et al. [50] licensed by CC BY.) (**D**) A microfluidic platform with integrated SPE for ApoE detection. (The image is reproduced from reference [51] with permission from Elsevier, copyright 2014.).

The SERS-based method offers a novel perspective for the detection of neurological diseases. For instance, Yuan et al. developed a microfluidic device for SERS immunoassay based on nanocellulose paper (NanoPADs) (Figure 3A), specifically designed for the ultrasensitive and cost-effective detection of the AD biomarker GFAP [48]. The research team created a SERS-active substrate by in situ synthesis of silver nanoparticles (AgNPs) on the surface of nanocellulose paper and combined it with gold NPs labeled with 5,5′-dithiobis-2-nitrobenzoic acid as a signal probe. By employing the ELISA method and optimizing experimental conditions, the researchers achieved ultrasensitive detection of GFAP with a detection limit as low as 150 fg/mL. This method not only boasts high sensitivity and low cost but also offers simplicity in operation, providing a robust technological tool for the early diagnosis of AD. Sun et al. also utilized SERS technology to develop a microcavity-based microfluidic chip (Figure 3B), which is capable of simultaneously quantitatively detecting two core biomarkers of AD: Aβ1-42 and p-Tau181 protein [49]. The research team employed a gas-assisted gas–liquid interface self-assembly technique to construct a uniform array of polystyrene (PS) microspheres. Subsequently, through oxygen plasma treatment and magnetron sputtering, gold nanoparticles (AuNPs) were successfully attached to these microspheres to enhance the local LSPR effect. To predict the electromagnetic field enhancement resulting from the optical resonance coupling between the PS microcavities and AuNPs, they employed finite-difference time-domain simulations, thereby forming SERS hot spots on the surface of the microspheres. Additionally, the researchers synthesized AuNPs modified with Raman reporter molecules and specific antibodies, creating dual-signal probes which were integrated into the SERS-active substrate of the microfluidic chip. Thanks to the design of the multi-channel microfluidic system, the chip can rapidly and sensitively detect Aβ1-42 and p-Tau181 proteins in AD, with an ultra-sensitive response as low as 100 fg/mL. Using commercially available portable Raman systems, it also enables early screening of AD patients at home.

Electrochemical detection is also a commonly used method, which involves measuring the current-voltage characteristics to detect biomarkers. Electrochemical detection can identify the electrochemical signals during the reaction process. It can significantly enhance detection sensitivity while ensuring specificity, allowing for detection even at low substance concentrations [79]. Moreover, electrochemical detection exhibits relatively low dependence on environmental factors such as light and temperature, allowing it to operate stably in more complex environments [80]. These characteristics have facilitated the expansion of its application range. Koklu et al. developed a microscale organic electrochemical transistor (OECT) integrated with a microfluidic platform (Figure 3C), which consists of a nanoporous membrane and microfluidic channels (μf-OECT), enabling label-free detection of Aβ aggregates in human serum [50]. A distinctive feature of this device is the presence of a nanoporous membrane functionalized with Congo red at the channel–electrolyte interface of the OECT, which exhibits strong affinity for Aβ aggregates. When Aβ aggregates bind to the membrane, they modulate the vertical ion flow to the channel, thereby altering the electrical properties of the transistor and achieving highly sensitive detection of Aβ aggregates. Notably, this detection method is independent of the ionic strength of the solution and does not involve chemical functionalization of electronic components, offering a novel perspective for the detection of Aβ aggregates. Medina-Sánchez et al. demonstrated a magnetic immunoassay method based on microfluidic devices for the detection of Apolipoprotein E (ApoE), a biomarker linked to AD [51]. The microfluidic chip developed in the study integrated screen-printed electrodes (SPE) (Figure 3D), which immobilized capture antibodies on magnetic microbeads to form immune complexes. Subsequently, biotin-labeled detection antibodies and streptavidin-quantum dots655 (Streptavidin-QD655) were employed as a signal amplification system. Quantum dots, also known as semiconductor nanocrystals, are nanoscale crystal clusters. Since each quantum dot can release multiple Cd^2+^ ions, these ions can be detected in electrochemical measurements, thereby significantly amplifying the signal. The electrochemical detection utilized square wave anodic stripping voltammetry, achieving a detection limit of 12.5 ng/mL and a linear range of 10–200 ng/mL. This approach developed a novel electrochemical method for detecting another biomarker of AD. In future research, enhancements in the microchannel design could focus on reducing incubation times and optimizing the reaction stage, potentially through the incorporation of mixers. Moreover, the sensitivity of electrochemical measurements could be improved by utilizing alternative nanomaterials for electrode surface modifications.

Microfluidic technology, combined with nanomaterials, has developed ultrasensitive detection methods for biomarkers of AD and other neurological disorders. These methods support label-free and highly sensitive detection requirements, significantly advancing the feasibility of early disease diagnosis and treatment. Future research is expected to delve deeper into the in vitro study of more biomarkers, aiming to discover additional early diagnostic markers for neurological diseases, thereby providing more comprehensive diagnostic support for clinical practice.

### 2.3. Detection of Cancer Biomarkers

Cancer is one of the leading causes of death worldwide. Most cancer patients have metastatic disease when first diagnosed [28], and the early symptoms of cancer are often non-specific, which undoubtedly increases the difficulty of diagnosis [81]. Therefore, early and effective detection of cancer biomarkers is crucial for diagnosis, treatment, and prognosis. Nanomaterial-integrated microfluidic technology has shown great potential in the detection of cancer biomarkers. This technology combines the precise control of microfluidic devices with the high sensitivity and specificity of nanomaterials, enabling rapid and highly sensitive detection. This not only meets the clinical demand for early cancer diagnosis but also supports the implementation of personalized treatment strategies.

#### 2.3.1. Detection of CTCs and ctDNA

Circulating tumor cells (CTCs) are shed from primary tumors and enter the peripheral blood circulation, playing a crucial role in the process of cancer metastasis [82]. In recent years, CTCs and circulating tumor DNA (ctDNA) have garnered significant attention as emerging biomarkers in translational research and clinical applications. Although these two biomarkers have been widely used in clinical diagnostics, their extremely low concentrations in the blood pose significant challenges for detection [83,84]. The advent of nanomaterial-enhanced microfluidic platforms has brought about major breakthroughs in the detection of CTCs and ctDNA, greatly advancing the field of cancer detection.

**Figure 4:**
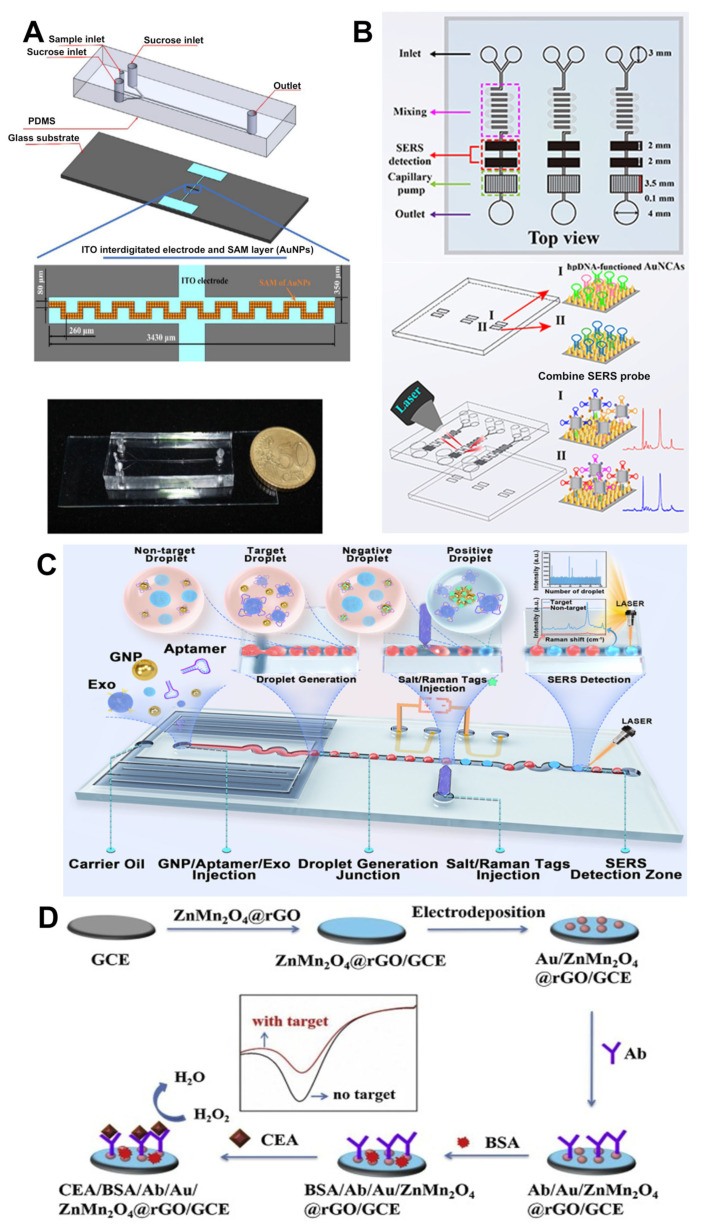
Detection of ctDNA and protein using NPs and microfluidic devices. (**A**) The microfluidic chip uses a combination of DEP technology and aptamers to capture A549 cells. The figure includes a schematic diagram of the chip and details of the configuration and dimensions of the interdigitated electrode, which is composed of indium tin oxide and AuNPs. (The image is reproduced from reference [52] with permission from John Wiley and Sons, copyright 2024.) (**B**) A novel SERS microfluidic chip designed for detecting ctDNA levels in serum following lung cancer treatment, utilizing functionalized AuNCAs as capture substrates. (The image is reproduced from Qian et al. [54] licensed by CC BY.) (**C**) Illustration of the process of SERS-based droplet microfluidic platform for detecting HER2-positive exosomes. (Reprinted with permission from [57]. Copyright 2024 American Chemical Society.) (**D**) The schematic diagram of the amperometric immunosensor preparation. (The image is reproduced from reference [61] with permission from Elsevier, copyright 2021.).

Lin et al. proposed a novel microfluidic chip (Figure 4A) for the efficient capture of A549 lung cancer cells [52]. This chip integrates dielectrophoresis (DEP) technology and aptamer binding, significantly enhancing capture efficiency and specificity by manipulating CTCs through a non-uniform electric field. The study compared three different chip designs: an unmodified glass surface, a glass surface modified solely with AuNPs, and a glass surface modified with both AuNPs and aptamers. The results demonstrated that AuNPs significantly improved capture efficiency under DEP, while the aptamer-functionalized surface maintained a CTC capture rate of over 80% in the absence of an electric field. Additionally, the study introduced a reverse pumping method that effectively mitigated inlet clogging, thereby enhancing experimental efficiency. These findings strongly underscore the importance of appropriate surface modifications in improving the efficiency of CTC capture in liquid biopsy using microfluidic chips, offering valuable technological tools for the early diagnosis and treatment of lung cancer. However, despite these advancements, CTC research continues to face persistent challenges, most notably the urgent need for a substantial increase in sensitivity.

Pedrosa et al. developed a CTCs detection system based on gold NPs and a microfluidic platform which can capture pancreatic cancer CTCs within 120 min [53]. In their study, the EpCAM antibody modified on the surface of AuNPs can specifically capture CTCs, significantly enhancing the capture efficiency of CTCs. Moreover, by optimizing the flow rate, it ensures that CTCs have sufficient time to bind with the antibodies while passing through the chip, further improving the detection sensitivity. The microfluidic chip was composed of a glass slide and a polydimethylsiloxane (PDMS) layer, fabricated using soft lithography. The experimental conditions, including flow rate and nanoparticle usage, were optimized, revealing that the capture efficiency of the nanoparticle-integrated lateral filter array exceeded 94% at a flow rate of 0.5 µL/s. Furthermore, the captured CTCs could be successfully released through trypsin treatment while maintaining high cell viability, making them suitable for subsequent cellular analysis. The use of NPs in the microfluidic device significantly reduced the analysis time for CTC capture and detection, enabling the processing of 4 mL of blood sample within 20 min. This method demonstrates rapid and efficient CTC capture capabilities, highlighting its significant potential for clinical applications.

Qian et al. developed a SERS-based microfluidic chip (Figure 4B) that integrates enzyme-assisted signal amplification and catalytic hairpin self-assembly signal amplification strategies for the detection of lung cancer-related ctDNA [54]. The chip utilizes a hairpin DNA-functionalized Au nanocone arrays (AuNCAs) as a capture platform, successfully enabling the simultaneous detection of four key prognostic ctDNAs in the serum of lung cancer mice. AuNCAs provide high-density hotspots that enhance the Raman signal. The method utilizes polymerase and nucleic acid endonuclease to exponentially amplify the target ctDNA, further enhancing the signal by forming stable complexes. This dual-signal amplification approach significantly improves the sensitivity and specificity of ctDNA detection. Furthermore, the detection results of the SERS microfluidic chip demonstrate reliability and exhibit a lower detection limit compared to traditional ELISA, making it suitable for real-time monitoring of ctDNA. These characteristics highlight the significant potential of the chip as a powerful clinical tool for assessing the efficacy of lung cancer treatment. Balakrishnan et al. also proposed a microfluidic platform-based method for ctDNA separation, aimed at early cancer detection [55]. The core of the study involves the use of superparamagnetic (SPM) bead particles on a microfluidic platform to extract and separate ctDNA. These bead particles carry a negative charge under conditions of low ionic strength and high acidity, enabling them to specifically bind to ctDNA. Through magnetic manipulation, the SPM bead particles can efficiently extract and separate ctDNA from plasma samples. The microfluidic chip designed in this study features multiple inlets and outlets, as well as curved microchannels, to facilitate sample mixing and flow. This design ensures sufficient contact between ctDNA and SPM beads, achieving efficient adsorption and separation of ctDNA, thereby improving the purity and yield of the separation. On average, 5.7 ng of ctDNA can be isolated from every 10 mL of plasma, with a detection sensitivity of 65.57% and a specificity of 95.38%. These results indicate that this method holds significant potential for early cancer detection and could provide crucial support for the early diagnosis of cancer. It is noteworthy that Huang et al. designed a specific nucleic acid microfluidic capture device based on DNA nanostructures (P-mesh) [56]. The DNA nanomesh is self-assembled from three rolling circle amplification amplified long chains, which are then treated with topoisomerase-I to form a stable topological structure combined with padlock probes. The microfluidic chip provides a closed environment that enhances the stability of the P-mesh, allowing it to maintain its structure and functionality for an extended period at room temperature. P-mesh, designed with specific nucleic acid capture sites, achieves high sequence specificity by selectively capturing ssDNA samples at a concentration as low as 1 pM and clearly distinguishing single-base mismatches. Compared to other methods, P-mesh enhances detection specificity at the molecular level, which is beneficial for the early detection of tumor ctDNA.

#### 2.3.2. Detection of Cancer-Related Extracellular Vesicles and Protein Biomarkers

Exosomes are a unique type of extracellular vesicles (EVs) which reflect tumor characteristics. Present in various biological fluids, exosomes are ideal candidates for liquid biopsy, enabling early cancer diagnosis, disease progression monitoring, and treatment efficacy evaluation [85]. Ho et al. developed an innovative droplet microfluidic platform integrated with a SERS-based aptasensor (Figure 4C), designed for rapid and sensitive detection of HER2-positive exosomes in breast cancer cells [57]. In the presence of HER2 aptamers and HER2-positive exosomes, salt-induced aggregation of GNPs on the chip enhances hotspot-based SERS signals. This platform achieves a detection time of just 5 min per sample. The technology has been successfully applied to clinical samples for HER2 detection, allowing for the accurate differentiation between HER2-positive and HER2-negative breast cancer patients. Compared to traditional exosome detection methods, this SERS-based droplet system significantly reduces detection time and holds potential for high-throughput screening of tumor exosomes, offering a non-invasive tool for early breast cancer diagnosis. Ortega et al. developed a microfluidic-based amperometric immunosensor for the detection of claudin7 (CLD7) in circulating EVs, a cancer biomarker used for colorectal cancer (CRC) diagnosis [58]. This innovative sensor employs synthetic MIL-125-NH2 (Materials Institute Lavoisier) NPs, which are covalently immobilized on the central channel of the microfluidic immunosensor. Using this nanomaterial as a platform, researchers achieved the immobilization of monoclonal antibodies specific to CLD7, enabling the specific recognition and capture of CLD7 in EV samples. Quantitative detection was performed using HRP-conjugated anti-CLD7 antibodies, generating a current signal proportional to the CLD7 levels in the sample. Compared to traditional ELISA methods, the total detection time of this amperometric immunosensor is significantly reduced, requiring less than 30 min. This research introduces novel prospects for utilizing porous open-framework platforms in biomedical and diagnostic sensing applications.

For the detection of protein biomarkers in CRC, Cao et al. developed a SERS platform based on a microfluidic chip [59]. Heterogeneous nuclear ribonucleoprotein A1 (hnRNPA1) and S100 calcium-binding protein P (S100P) are among the most significant alteration-induced proteins in CRC, and the enhanced expression of both is closely associated with the progression of CRC. The platform employs a novel Au nanocrown array (AuNCA) to achieve highly sensitive detection of these two protein biomarkers, hnRNPA1 and S100P, through a one-step recognition-release mechanism. The detection process can be completed within 15 min, with detection limits reaching 0.031 pg/mL and 0.057 pg/mL, respectively. Furthermore, the platform’s detection results are consistent with those of ELISA, demonstrating good clinical testing consistency. The study highlights the platform’s potential for early CRC diagnosis, as it enables simultaneous rapid detection of multiple biomarkers compared to ELISA, particularly in scenarios requiring fast and sensitive detection. This is of significant importance for improving the early diagnosis rate of CRC and the survival rate of patients.

Matrix metalloproteinase-9 (MMP-9) and IL-6 are also critical factors in tumorigenesis. MMP-9 can degrade components of the extracellular matrix and is highly expressed in gastric cancer. Huang et al. successfully developed a sensing system based on microfluidic technology and SERS for the highly sensitive and specific detection of gastric cancer biomarkers MMP-9 and IL-6 [60]. The study utilized gold-coated iron oxide NPs (Fe_3_O_4_@AuNPs) and gold nanocages (AuNCs) as SERS-active substrates, employing an aptamer recognition release mechanism and a dual signal amplification strategy to enhance the detection signal. The magnetization and enrichment of the complexes were achieved through the application of a magnetic field on the microfluidic chip, further enhancing the SERS signal and enabling highly specific and ultrasensitive detection of the target molecules. The detection limits for MMP-9 and IL-6 were as low as 0.178 pg/mL and 0.165 pg/mL, respectively, which are significantly lower than those of traditional ELISA. The system exhibits significant promise for the early detection of gastric cancer.

Carcinoembryonic antigen (CEA) is a cell surface glycoprotein that is significantly overexpressed in various common cancers. The levels of CEA can indicate tumor survival and progression and are closely associated with patient survival rates [86]. Fan et al. developed a novel amperometric immunosensor (Figure 4D) for the detection of CEA [61]. This sensor utilizes the electrocatalytic activity of ZnMn_2_O_4_@reduced graphene oxide (ZnMn_2_O_4_@rGO) composite material towards hydrogen peroxide to amplify the immune response signal, enabling highly sensitive detection of CEA. Due to the excellent biocompatibility and bioconjugation capabilities of gold NPs, which provide a stable interface for immune reactions, Fan et al. used gold nanoparticle-modified ZnMn_2_O_4_@rGO composite material to immobilize antibodies. This allows the sensor to successfully detect CEA within a wide linear range (0.01 to 50 ng/mL) and a low detection limit (1.93 pg/mL), demonstrating excellent sensitivity and specificity. The overall detection time is superior to traditional detection methods, making it particularly suitable for the early diagnosis and monitoring of clinical cancers.

In the field of tumor biomarker detection, the integration of nanomaterials with microfluidic technology is demonstrating significant advancements. With its high sensitivity and specificity, this technology not only facilitates early cancer diagnosis but also supports the implementation of personalized treatment strategies. Microfluidics combined with nanomaterials can detect cancer-related extracellular vesicles and protein biomarkers, allowing for the early identification of cancer patients and differentiation among subtypes of different cancer patients, thereby enabling the development of targeted treatment strategies [87]. Moreover, this technology can monitor changes in biomarkers during the treatment process, allowing for timely adjustments to the therapeutic regimen, ensuring sustained treatment effectiveness. It is expected that future research will focus on enhanced detection of multiple cancer markers and optimization of methods, thus broadening the scope of this technology in early cancer diagnosis and treatment.

We compared the detection technologies applied in various diseases diagnoses mentioned above and conducted an in-depth analysis of the advantages and disadvantages of different detection techniques (Table 2).

## 3. The Integration of Nanomaterials with Microfluidic Technology for Disease Treatment

Nanomaterials have shown significant potential in the early diagnosis and treatment of diseases. Through their capacity to amplify detection signals or act as substrates for capturing biomarkers, nanomaterials have greatly progressed early disease diagnosis, with profound implications for enhancing cure rates and reducing treatment costs. In disease treatment, nanomaterials are frequently utilized as effective drug carriers (Table 3), enhancing both the biocompatibility of drugs and their therapeutic effectiveness.

Nanomedicine refers to innovative drug systems created and engineered using nanotechnology for the purpose of diagnosing, treating, or preventing diseases. These drug systems exhibit advantages due to their unique physicochemical properties, such as high surface area, tunable size, and shape, which enhance drug delivery efficiency, improve targeting, and reduce side effects [89,90,91]. Microfluidic technology has provided essential tools for in vitro studies of the therapeutic effects of nanomedicine. These models overcome the limitations of traditional two-dimensional cell cultures and animal models, which fail to fully simulate the complex microenvironment of the human body, thereby significantly improving the accuracy of drug screening and the reliability of efficacy evaluation [92,93,94].

**Table 3:** Summary of nanomaterials in disease treatment applications.

Nanomaterial Name	Size	Shape	Function	References
RGN ps, CGNps	3 nm/20 nm, 20 nm	sphere	reducing oxidative stress in endothelial cells	[95]
pNPs	20 nm	sphere	researching vascular barrier permeability	[96]
GCPIH	263 nm	sphere	enhancing thrombolytic effects	[97]
tPA-DPNs	1000 nm of diameter, 400 nm of height	discoidal	improving thrombolytic efficiency	[98]
Tf@pSiNPs, BSA@pSiNPs	182 ± 1 nm, 174 ± 1 nm	sphere	enhancing the permeability of BBB	[99]
angiopep-2 functionalized lipid cubosomes	300 nm	cubic phase	encapsulating TMZ and CDDP, treating GBM	[37]
multiple NPs	N/D	sphere	treating GBM	[100]
AuNPs@POM@PEG	17.7 ± 2.3 nm	sphere	inhibiting the aggregation of β- amyloid	[101]
D-T7/Tet1-lipids@PL	68.93 ± 0.59 nm	core-shell structure	delivering LTG, treating epilepsy	[102]
DTXL-SPN	N/D	sphere	as a carrier of DTXL	[103]
IMQ-HA-GEM	52.4 nm	sphere	delivering GEM and IMQ, enhancing therapeutic efficacy	[104]
HGNs@anti-MUC1	N/D	spherical hollow structure	as a photothermal agent, photothermally treating tumors	[105]
PEG-liposomes, PEG-PLGA NPs	70 nm	sphere	as a drug carrier	[106]
PTX-PLGA-SH NPs	133.6 ± 2.1 nm	sphere	encapsulating and delivering PTX	[107]

RGNps, resveratrol gold nanoparticles; CGNps, citrate gold nanoparticles; pNPs, polystyrene nanoparticles; GCPIH, Glycol chitosan-polypyrrole-iron oxide-heparin; tPA, tissue plasminogen activator; tPA-DPNs, tpa-discoidal polymeric nanoconstructs; Tf@pSiNPs, Transferrin-functionalized porous silicon nanoparticles; BSA@pSiNPs, Bovine Serum Albumin-functionalized porous silicon; BBB, blood–brain barrier; TMZ, temozolomide; CDDP, cisplatin; GBM, glioblastoma multiforme; N/D, not determined; AuNPs, gold nanoparticles; POM, polyoxometalates; PEG, polyethylene glycol; D-T7/Tet1-lipids@PL, D-T7/Tet1-lipids@PLGA-Lamotrigine nanoparticles; LTG, lamotrigine; DTXL-SPN, docetaxel-spherical polymeric nanoparticles; DTXL, docetaxel; IMQ-HA-GEM, hyaluronic acid-gemcitabine-imiquimod; GEM, gemcitabine; IMQ, imiquimod; HGNs@anti-MUC1, hollow gold nanoshells@anti-MUC1 aptamer; PEG-PLGA NPs, poly(ethylene glycol)/poly(lactide-co-glycolic acid) nanoparticles; PTX, paclitaxel; PTX-PLGA-SH NPs, PTX-loaded nanoparticles.

### 3.1. Vascular on a Chip

Nanoparticle drug delivery systems offer advantages in improving drug efficacy, minimizing side effects, and enabling targeted therapy. Nanomaterials can improve the targeted delivery and penetration capabilities of drugs, serving as drug carriers to enhance drug permeability [108]. Additionally, nanomaterials can modify drug properties, thereby improving drug utilization rates [109]. Microfluidic vascular chips can accurately mimic the vascular microenvironment and replicate the hemodynamic characteristics of blood flow, enabling more precise studies of phenomena such as thrombosis, platelet adhesion, and aggregation [110]. These chips offer a robust tool for examining the interactions between blood cells and nanoparticle drugs, as well as investigating critical factors affecting the accumulation of nanoparticle drugs in blood vessels. Additionally, such chips can evaluate the influence of vascular permeability on the transport of nanoparticle drugs and assess the therapeutic effectiveness of nanoparticle drugs [111]. Traditional cell culture models are unable to simulate the transport process of drugs across the endothelial cell barrier in vivo, which is crucial for the pharmacokinetics of drugs. The use of fluid-coupled multi-organ chip models effectively addresses this challenge. Computational modeling can transform in vitro data of clinical drugs obtained from multi-organ chip models into in vivo predictions, and the final in vivo predictions closely resemble clinical data [112].

**Figure 5:**
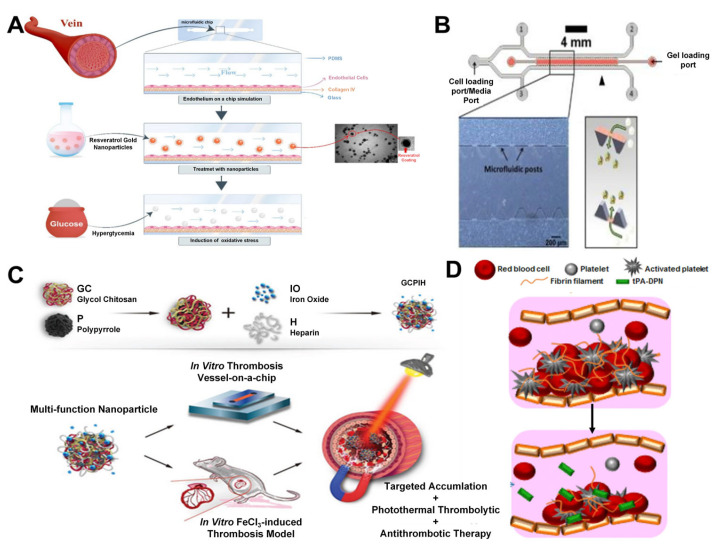
Nanomaterials in disease treatment within microfluidic-constructed vascular chips. (**A**) Investigating the antioxidant effects of RGNps in microfluidic vascular chips. The figure includes a schematic diagram of the vascular chip and the induction of oxidative stress using glucose and the evaluation of the antioxidant properties of RGNps within a microchannel simulating a vein. (The image is reproduced from Fayazbakhsh et al. [95], licensed by CC BY.) (**B**) Microfluidic devices for evaluating the permeability of in vitro barriers to NPs. The figure includes cross-sections of untreated cells cultured in a microfluidic device for four days and the diffusion interface of NPs. (The image is reproduced from Ho et al. [96], licensed by CC BY.) (**C**) Microfluidic vascular chip uses multiarm self-indicating nanoassembly for photothermal-enhanced thrombolytic therapy. The figure includes schematic diagrams of multiarm self-indicating nanoassembly synthesis and FeCl_3_-induced animal thrombosis and microfluidic vascular chip models used for photothermal thrombolysis. (The image is reproduced from reference [97] with permission from John Wiley and Sons, copyright 2023.) (**D**) Nanotherapeutics utilizing microfluidic chips to simulate blood vessels involves directly combining a clinical formulation of tPA with the porous structure of soft discoidal polymeric nanoconstructs. (Reprinted with permission from [98]. Copyright 2018 American Chemical Society.).

Fayazbakhsh et al. designed a microfluidic chip (MFC) (Figure 5A) to simulate blood vessels and investigate the antioxidant properties of resveratrol gold nanoparticles (RGNps), citrate gold nanoparticles (CGNps), and free resveratrol on human umbilical vein endothelial cells (HUVECs) [95]. The study constructed a single-channel MFC by modulating collagen levels to culture endothelial cells. These endothelial cells cultured in the MFC responded to dynamic flow, exhibiting spindle-shaped and stretched morphologies that were markedly different from those under static culture conditions. Consequently, the MFC provided a more precise and reliable platform to promote the natural morphology and functional performance of cells. GNps were employed as carriers for resveratrol to enhance its bioavailability, as they can inhibit the generation of reactive oxygen species (ROS), suppress free radicals, and increase antioxidant-active defense enzymes in the body. The study demonstrated that in the vascular environment simulated by the MFC, 20 nm RGNps reduced high glucose-induced endothelial oxidative stress by 57–82%, outperforming both CGNps and free resveratrol, thereby exhibiting significant antioxidant properties.

Ho et al. developed an in vitro model based on microfluidic technology for the precise measurement of nanoparticle diffusion permeability coefficients (Figure 5B) [96]. By modulating the permeability of human umbilical vein endothelial cells, Ho et al. successfully simulated both healthy and tumor vascular environments, thereby investigating the accumulation and penetration characteristics of polystyrene nanoparticles (pNPs). The research findings revealed that in the simulated tumor vascular environment, smaller-sized pNPs exhibited higher permeability, while larger-sized pNPs showed relatively lower permeability. The successful establishment of this model contributes to offering additional insights for the study of thrombolytic therapy on vascular chips, thereby delving into the thrombolytic effects of a broader range of nanomedicines.

Liu et al. developed a microfluidic model capable of predicting thrombolysis kinetics (Figure 5C), which was used to evaluate the targeting ability of glycol chitosan-polypyrrole-iron oxide-heparin (GCPIH) NPs in accumulating at thrombus sites and their photothermal thrombolysis effects under simulated physiological blood flow conditions [97]. The bionic multi-arm self-indicating nanoassembly they designed can achieve precise delivery of thrombus, photothermal-enhanced thrombolysis, and anticoagulation therapy. They used the photolithography technology to fabricate a microfluidic chip and seeded HUVEC in the chip to form a vascular-like structure. Endothelial cells were stimulated with lipopolysaccharide, followed by perfusion of human whole blood to induce thrombus formation. Subsequently, Cy5-labeled GCPIH were perfused into the vascular chip to assess their accumulation in the thrombus microenvironment. The chip was then exposed to a near-infrared laser to monitor temperature fluctuations, evaluate the photothermal properties of the GCPIH post-laser exposure, and examine changes in fibrin clots to determine the thrombolytic efficacy of the GCPIH. By simulating the complex processes of in vivo blood flow and thrombus formation, this model provides valuable insights for the development and optimization of thrombus treatment strategies.

Tissue plasminogen activator (tPA) is the only approved drug for the treatment of acute ischemic stroke. However, due to medical contraindications and severe side effects, such as cerebral hemorrhage, only a small number of patients can benefit from this treatment. Colasuonno et al. developed a novel nanotherapeutic agent by combining tPA with the porous structure of soft discoidal polymeric nanoconstructs (tPA-DPNs). They utilized a microfluidic chip to simulate the in vivo environment of clot formation, providing a dynamic platform for studying the thrombolytic efficacy of tPA-DPNs (Figure 5D) [98]. In the microfluidic chip, the thrombolytic efficiency of tPA-DPNs and free tPA was compared. The results showed that tPA-DPNs reduced the clot area by half within 60 min, whereas free tPA required 90 min, demonstrating the significant advantage of tPA-DPNs. Furthermore, 2.5 mL/kg of tPA-DPNs dissolved 90% of the thrombus, while the same dose of free tPA only dissolved 40%. tPA-DPNs can exert a more concentrated thrombolytic effect at the thrombus site without increasing the risk of systemic hemorrhage, thereby avoiding bleeding caused by excessive dosage. The microfluidic chip, as an experimental model, validated the effectiveness of tPA-DPNs as a novel nanotherapeutic agent in a simulated in vivo environment and provided critical data for its potential in clinical applications.

Microfluidic vascular chips, by simulating the blood vessel microenvironment, offer an in vitro model for investigating the interactions between nanomedicines and blood cells, as well as evaluating the influence of vascular permeability on the transportation of nanomedicines. This chip has evolved into a powerful tool for assessing the utility of nanomedicines in treating vascular conditions. However, different drugs exhibit distinct properties and liver absorption rates, leading to variations in drug absorption [112]. Future work will require further optimization of the physiological parameters in computer models to enhance the accuracy of translating in vitro data from different drugs into in vivo predictions.

### 3.2. Blood–Brain Barrier Chip

The blood–brain barrier (BBB) is a specialized barrier between the circulatory system and neural tissue. It plays a crucial role in regulating the transport of substances essential for brain function and protecting the brain from harmful substances in the blood [113,114]. The BBB consists of closely packed endothelial cells with tight junctions that greatly limit the penetration of macromolecules and NPs. Additionally, the BBB includes a basement membrane and a layer of pericytes, which further increase the complexity of the physical barrier [115,116,117]. NPs increase drug solubility through size reduction, enhancing their capability to traverse the BBB [24]. Nanocarriers can be modified with particular ligands or antibodies, allowing them to selectively identify and attach to receptors on the BBB, thereby aiding in the targeted absorption of drugs [118]. BBB models utilizing microfluidic technology play a vital role in assessing the penetration and therapeutic impact of nanomedicines across the BBB in in vitro research focused on the BBB.

**Figure 6:**
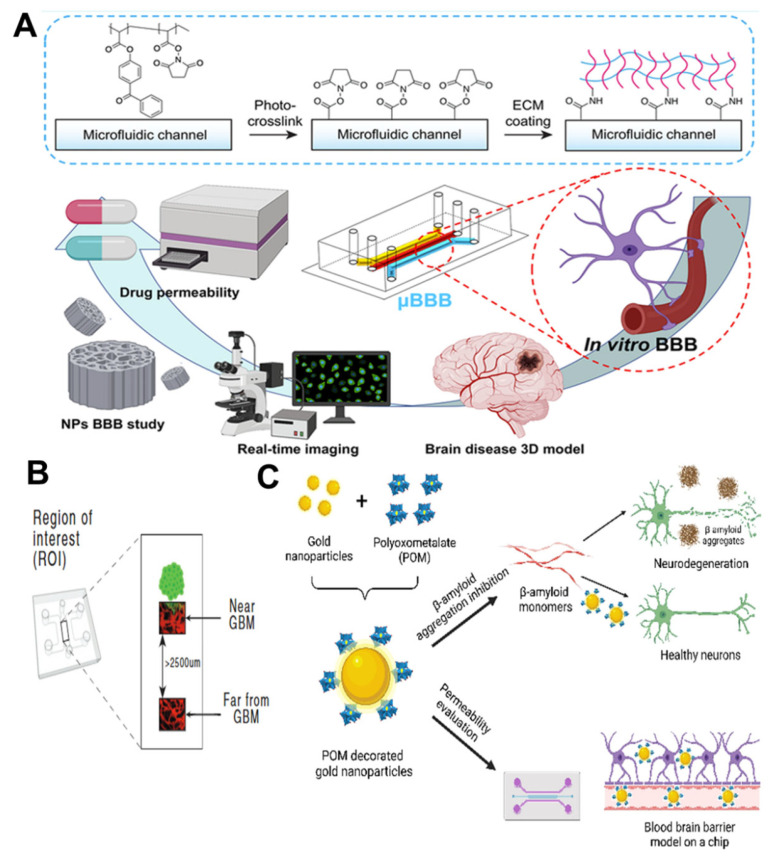
Treatment of diseases using nanomaterials via microfluidic-constructed blood–brain barrier chips. (**A**) Schematic diagram of μBBB manufacturing and nanomaterial detection process. The figure includes the process of fabricating μBBB platform by photo-crosslinking a copolymer within microfluidic channels, followed by extracellular matrix coating, and its applications in drug permeability testing, real-time nanoparticle tracking, and 3D brain disease modeling. (Reprinted with permission from [99]. Copyright 2020 American Chemical Society.) (**B**) Region of interest identified spatially within the BBB-GBM model. (The image is reproduced from Straehla et al. [100], licensed by CC BY.) (**C**) Schematic diagram of AuNPs@POM@PEG synthesis and inhibition of Aβ, and evaluation of permeability using BBB-on-a-chip. (The image is reproduced from Perich et al. [101], licensed by CC BY.).

Peng et al. developed a microfluidic model (μBBB) (Figure 6A) to simulate the BBB for predicting the brain uptake of central nervous system drugs and NPs (Transferrin-functionalized porous silicon NPs (Tf@pSiNPs) and Bovine Serum Albumin-functionalized porous silicon NPs (BSA@pSiNPs)) [99]. This model utilizes a photocrosslinkable copolymer containing specific functional groups to achieve uniform coating and functionalization of the microfluidic channels, forming a stable layer of extracellular matrix proteins. By employing this photocrosslinkable copolymer with specific functional groups, the model enables the in situ even coating and functionalization of μBBB chip channels, facilitating the co-culture of human endothelial cells, pericytes, and astrocytes, thereby accurately simulating the BBB. The model exhibited exceptional accuracy in predicting the uptake of small molecules and NPs in brain tissue. The permeability coefficients of the tested compounds aligned well with established research findings, further validating the model’s credibility. Additionally, the model allows for the direct observation of nanoparticle transport through receptor-mediated endocytosis, providing a multifunctional platform that integrates prediction and visualization for brain disease research.

The blood–brain barrier model for tumors is also a crucial direction in in vitro research. Cai et al. utilized microfluidic technology to construct a BBB/GBM-on-a-chip model, which accurately simulates the complex microenvironment of the BBB and glioblastoma multiforme (GBM). This innovative model is of great significance for advancing the development of drug delivery systems tailored to target GBM tumor sites directly [37]. The newly developed nanodrug delivery system—angiopep-2 functionalized lipid cubosomes—was designed to carry cisplatin (CDDP) and temozolomide (TMZ), commonly used in GBM treatment. Experimental results demonstrated that these angiopep-2-functionalized lipid cubosomes exhibited higher efficiency in penetrating the BBB, highlighting the potential of microfluidic technology in evaluating and optimizing nanoparticle targeting. In another study related to GBM, Straehla et al. also constructed a microfluidic model simulating GBM (Figure 6B) and assessed the ability of NPs to penetrate the BBB [100]. By employing in vivo imaging techniques, they directly observed and measured NPs and fluorescein within the brain capillaries of mice and compared the BBB permeability of NPs between the in vitro microfluidic model and the actual mouse model. After comparing the data obtained in vivo with the in vitro model data, researchers found a robust consistency in the BBB permeability of NPs and fluorescein. Additionally, they evaluated the efficacy of cisplatin-loaded functionalized NPs in both in vitro and in vivo models. The results demonstrated that, compared to non-functionalized NPs, functionalized NPs exhibited more abundant accumulation in GBM tumors and showed superior therapeutic effects in both in vitro and in vivo models. These findings not only advance GBM treatment research but also provide new directions for the application of nanomedicine in GBM therapy.

Perich et al. evaluated the newly developed tri-component nanohybrid system, which is based on AuNPs and surface-modified with polyethylene glycol (PEG) and polyoxometalates (POM), using the BBB-on-a-chip technology (Figure 6C) [101]. The BBB-on-a-chip system is composed of a 3D fibrin hydrogel embedded with human astrocytes and pericytes, forming a brain compartment that directly interfaces with human endothelial cells in the blood channel. Through the BBB-on-a-chip model, the researchers assessed the cytotoxicity of AuNPs@POM@PEG and its ability to cross the BBB. The experimental results demonstrated that AuNPs@POM@PEG exhibited low cytotoxicity in the BBB-on-a-chip model and effectively penetrated the BBB. This finding holds significant implications for the development of novel therapies targeting the inhibition of Aβ aggregation in AD. For the treatment of epilepsy, Hou et al. utilized microfluidic technology to generate double targeting nanoparticle-D-T7/Tet1-lipids@PLGA-Lamotrigine nanoparticles (D-T7/Tet1-lipids@PL), loaded with lamotrigine (LTG), and investigated their therapeutic efficacy for epilepsy using a microfluidic-based BBB model [102]. The D-T7 peptide, by binding to the transferrin receptor, can guide the drug across the BBB, while the Tet1 peptide specifically binds to receptors on the neuronal surface, enabling efficient drug accumulation in neurons. D-T7/Tet1-lipids@PL also reduces the required drug dosage and mitigates the side effects associated with LTG. Results from the in vitro microfluidic BBB model demonstrated that D-T7/Tet1-lipids@PL exhibits favorable accumulation and penetration depth. Combined with in vivo experiments in mice, the feasibility of D-T7/Tet1-lipids@PL as a therapeutic agent for epilepsy was validated.

In summary, the microfluidic-based BBB model is essential for in vitro investigations of nanomedicines, enabling the assessment of their BBB penetration capabilities. This model is particularly crucial for advancing in vitro studies on nanomedicine therapies for neurological conditions by mimicking the BBB construction while minimizing reliance on live animals. Future research will focus on assessing the efficacy of nanomedicines in traversing the BBB, leading to enhanced drug delivery approaches for brain disease treatment.

### 3.3. Tumor on a Chip

Nanomaterials can serve as effective carriers for anticancer drugs, enhancing their specific binding to tumor cells through drug modification [119]. They can also increase drug loading capacity and promote drug release and absorption [120]. Microfluidic technology can accurately simulate the complex physiological environment within the human body, thereby constructing tumor models that more closely resemble real conditions. These models not only replicate the three-dimensional structure of tumor tissues but also simulate the tumor microenvironment, including angiogenesis and cell–cell interactions [93,100]. These microfluidic-based tumor models are designed for assessing the efficacy of nanomedicines in tumor treatment.

**Figure 7:**
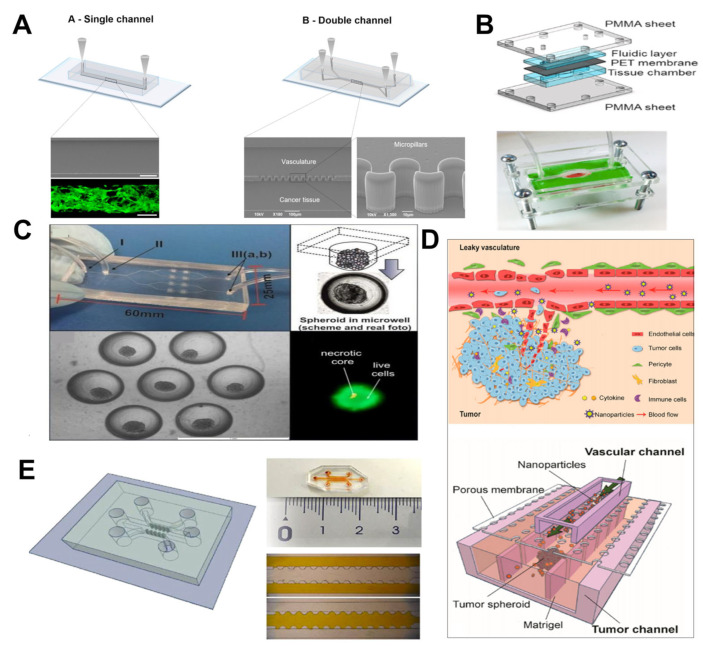
Evaluating the therapeutic effects of nanomaterials on tumors using microfluidic tumor chips. (**A**) Schematic diagram of single–channel and double–channel microfluidic tumor chips used to study the therapeutic effects of DTXL-SPN on U87-MG. (The image is reproduced from reference [103] with permission from Elsevier, copyright 2023.) (**B**) BCC evaluates the therapeutic efficacy of IMQ-HA-GEM on breast cancer. The figure includes schematic illustration of the layers of the 3D compartmentalized BCC device and photograph of the assembled BCC device. (The image is reproduced from reference [104] with permission from Elsevier, copyright 2025.) (**C**) Microfluidic tumor chip utilizing HGNs@anti-MUC1 for photothermal therapy. The figure includes images of a microsystem with inlet, outlet, and exhaust port and images of tumor spheroids and chambers for culturing spheroids and stained spheroids. (The image is reproduced from reference [105] with permission from Elsevier, copyright 2019.) (**D**) Schematic of the in vivo tumor microenvironment consisting of leaky vasculature and tumor tissues and schematic illustration of the in vitro TVOC model. (Reprinted with permission from reference [106]. Copyright 2018 American Chemical Society.) (**E**) Schematic illustration of a microfluidic device and the loading of collagen gel mixed with red food dye into the side and middle channels. (Reprinted with permission from reference [107]. Copyright 2020 American Chemical Society.).

Martins et al. fabricated single-channel and dual-channel microfluidic devices (Figure 7A) to simulate human glioblastoma multiforme (U87-MG) and investigated the effects of free docetaxel (DTXL) and docetaxel-loaded spherical polymeric nanoparticles (DTXL-SPN) on this model [103]. Martins et al. dissolved poly(lactic-co-glycolic acid) and DTXL in chloroform, mixed them with an aqueous phospholipid phase, and ultrasonicated the mixture to form an emulsion, followed by solvent evaporation under reduced pressure to prepare SPNs [121]. SPNs can form a stable shell that effectively encapsulates DTXL, enhancing the stability and solubility of the drug. This facilitates the efficient delivery of the drug within the body, reducing its degradation and clearance in the bloodstream. The single-channel and dual-channel devices simulated different drug delivery methods, respectively, while the interconnected parallel channels through microcolumns emulated the interplay between blood vessels and cancer tissues. DTXL-SPN was able to penetrate the micropillar membrane in the microfluidic device and diffuse within the tumor matrix. The survival rate of DTXL-SPN tumor cells decreased steadily with time. The feasibility of the device as a tool for predicting the efficacy of chemotherapy in U87-MG cells and the effectiveness of DTXL-SPN in tumor treatment has been validated.

Singh et al. developed a novel nanoparticle based on hyaluronic acid (HA), named IMQ-HA-GEM, which combines two drugs, gemcitabine (GEM) and imiquimod (IMQ). Utilizing microfluidic technology, they constructed a three-dimensional, compartmentalized breast cancer microfluidic model (Figure 7B) to simulate the tumor microenvironment and evaluate the penetration capability of the drug-loaded NPs [104]. The model consists of a top fluid layer, a bottom tissue chamber, and a porous PET membrane in between. Breast cancer cells were embedded in a gelatin methacryloyl (GelMA) hydrogel within the bottom tissue chamber. HA-drug NPs were circulated through the microfluidic channels to mimic the distribution and cellular uptake of the drugs in the tumor microenvironment. The experimental results demonstrated that IMQ-HA-GEM NPs significantly enhanced the infiltration of immune cells into breast cancer tissue compared to HA alone or HA-GEM NPs. This enhanced infiltration suggests the role of IMQ in attracting and promoting the migration of immune cells into the tumor microenvironment. When IMQ is combined with HA and GEM to form IMQ-HA-GEM NPs, it may activate immune cells by binding to specific receptors on immune cells, such as Toll-like receptors, thereby promoting the release of inflammatory factors and enhancing the immune response. Furthermore, the NPs demonstrated excellent cellular uptake capabilities and exhibited significant toxicity toward breast cancer cells. These findings confirm that IMQ-HA-GEM NPs enhance the anticancer efficacy of GEM by activating immune cells, providing a new direction for the development of breast cancer treatment strategies.

Mucin1 (MUC1) is a transmembrane glycoprotein that is upregulated in a range of epithelial-derived cancers including breast cancer, lung cancer, and pancreatic cancer. Kalinowska et al. synthesized hollow gold nanoshells (HGNs) modified with an anti-MUC1 aptamer (HGNs@anti-MUC1) for photothermal therapy (PTT) [105]. HGNs exhibit absorption characteristics in the near-infrared region and locally generate high temperatures, thereby enabling PTT, which is particularly suitable for targeted photothermal treatment of tumor cells. A microfluidic system was employed to construct and culture 3D multicellular spheroids as models for early-stage breast and lung tumors, simulating the necrotic core and the peripheral proliferative cell layer of the tumors (Figure 7C). The bottom layer of the microfluidic chip was designed with microchannels and chambers, where the microchannels were used to introduce cell suspensions, and the chambers were utilized for the formation and cultivation of spheroids. Experimental results demonstrated that these NPs, guided by specific aptamers, could selectively accumulate in MUC1-overexpressing tumor cells, generating localized hyperthermia under near-infrared radiation, effectively reducing the viability and size of the tumor spheroids. Compared to traditional photodynamic therapy, the near-infrared light used in PTT possesses greater tissue penetration depth, enabling PTT to treat deeper-seated tumors.

In addressing ovarian tumors, Wang et al. developed a microfluidic tumor vasculature-on-a-chip (TVOC) (Figure 7D) to simulate the leaky characteristics of tumor vasculature and the dense extracellular matrix (ECM) of 3D ovarian tumor tissue, aiming to investigate the passage and accumulation of NPs through tumor vasculature and within tumors [106]. The top channel was used to culture HUVECs to form vascular structures, which were then treated with tumor necrosis factor-α (TNF-α) to mimic the leaky properties of tumor vasculature. The bottom channel was utilized to culture tumor spheroids embedded in Matrigel to simulate tumor tissue. Two types of NPs, PEG-liposomes and poly(ethylene glycol)/poly(lactide-co-glycolic acid) nanoparticles (PEG-PLGA NPs), were prepared using microfluidic technology. Wang et al. also employed folic acid (FA) as a ligand to modify both types of NPs, enhancing their tumor-targeting capabilities. The results indicated that their transport through the vascular barrier and tumor ECM was significantly affected, while FA slightly improved the accumulation of PEG-PLGA NPs in the tumor model. The findings from the TVOC model were consistent with those from in vivo human ovarian cancer xenograft tumor models, confirming its effectiveness in predicting nanoparticle tumor accumulation and providing a robust in vitro evaluation tool for the development of nanomedicine delivery systems. Similarly, Wang et al. developed an ovarian-tumor-microenvironment-on-a-chip (TMOC) (Figure 7E) model and evaluated the effects of paclitaxel (PTX)-loaded nanomaterials [107]. The model utilized a microfluidic device made of PDMS material, featuring a central channel and two side channels, with the central channel filled with collagen gel to simulate tumor tissue. By dissolving PTX, PEG-PLGA, and shellac in a mixed solvent of ethanol, DMSO, and DMF, followed by mixing with PBS and using a precipitation method, PTX-loaded nanoparticles (PTX-PLGA-SH NPs) were prepared. These NPs composed of PLGA and shellac can protect PTX from degradation, enhance its bioavailability, and control its release. Furthermore, even low doses of PTX-PLGA-SH NPs could effectively kill tumor cells, with most of the drug released within the first 24 h, showing a dose-dependent behavior. The TMOC model provides a robust in vitro platform for evaluating nanomedicine delivery systems.

Microfluidic technology has effectively replicated the tumor microenvironment by developing three-dimensional models that mimic tumor tissues, incorporating crucial elements like angiogenesis and intercellular interactions. These models serve as a reliable experimental platform for evaluating the effectiveness of nanomedicines in treating tumors. In the future, research is expected to focus more on screening and optimization of nanomedicines to achieve more accurate targeted therapy for tumors, ultimately improving treatment efficacy and minimizing side effects.

## 4. Novel Medical Applications of Microfluidic Technology Integrated with Nanomaterials

Future advancements in the medical industry are heavily reliant on new medical technologies. The integration of nanomaterials and microfluidic devices has emerged as a promising frontier in this domain, driving the evolution of medical technology [122,123] and providing new possibilities for future medical solutions. Subsequently, this section will explore the application of nanomaterials (Table 4) and microfluidic devices in skin-interactive devices and imaging technology.

### 4.1. Skin-Interfacing Devices

Skin-interfacing devices refer to advanced technological apparatuses that can directly interact with human skin, enabling data exchange and sensory functions. These devices are typically characterized by their flexibility, lightweight nature, and wearability, allowing for real-time monitoring of physiological indicators or providing tactile feedback without disrupting daily activities [132,133]. Their design aims to seamlessly integrate into users’ daily lives while offering precise health monitoring and interactive experiences.

**Figure 8:**
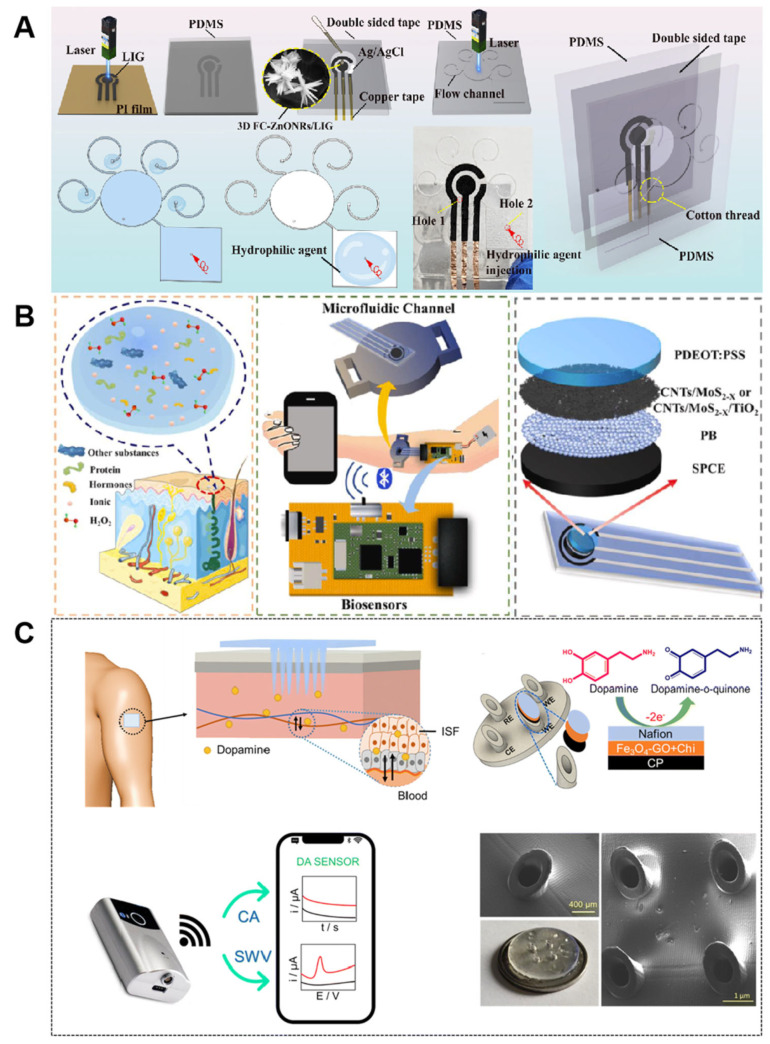
Skin interactive device for monitoring disease biomarkers using microfluidic technology combined with nanomaterials. (**A**) Schematic diagram of the fabrication of a microfluidic chemosensor for monitoring copper ions. The figure delineates the fabrication sequence of a microfluidic electrochemical sensor, encompassing the creation of graphene electrodes via LIG and the application of FC-ZnONRs modified on the LIG. Additionally, it includes the employment of hydrophilic agents to facilitate the autonomous flow of electrolyte within the microfluidic channels. (The image is reproduced from reference [124] with permission from Elsevier, copyright 2024.) (**B**) Schematic diagram of human skin and its sweat composition and schematic construction of a flexible microfluidic electrochemical sensor and schematic diagram of the composition of the sensor. (The image is reproduced from reference [125] with permission from Elsevier, copyright 2024.) (**C**) The figure presents a schematic representation of the microneedle sensor inserted into the skin to monitor dopamine levels in interstitial fluid, illustrates the dopamine detection mechanism involving dopamine-o-quinone formation at the electrode surface, depicts the wireless setup for continuous monitoring of dopamine using a smartphone to receive data from the sensor, and shows field emission scanning electron microscopy images for the morphology of the microneedle electrodes. (The image is reproduced from M. R. et al. [129] licensed by CC BY.).

Most skin-wearable devices integrated with microfluidic technology extract analytes from sweat or interstitial fluid. The electrochemical detection method is the most commonly used technique in skin microfluidic devices. Target substances undergo redox reactions on the electrode surface, generating measurable current or voltage signals, thereby enabling quantitative detection. Abnormal copper ion levels are associated with Wilson’s Disease and Menkes Disease, both of which are genetic disorders caused by copper metabolism dysfunction. Zhang et al. designed a microfluidic-based wearable chemical sensor for monitoring Cu^2+^ in sweat (Figure 8A) [124]. The sensor utilizes surface tension gradient-driven electrolyte flow, combined with a 3D composite material formed by laser-induced graphene (LIG) and flower cluster-shaped zinc oxide nanorods (FC-ZnONRs). LIG has a porous structure, providing numerous active sites, and the 3D FC-ZnONRs/LIG composite not only increases the specific surface area and active sites of the electrode but also enhances electron transport capability. This composite material enables the sensor to achieve a low detection limit (0.0368 μg L^−1^) and high sensitivity (0.414 μA (μg L^−1^) ^−1^ cm^−2^), allowing for the detection of extremely low concentrations of Cu^2+^, making it suitable for early disease diagnosis and environmental monitoring. In another study on monitoring biomarkers in sweat, Ying et al. developed a microfluidic wearable electrochemical sensor for non-invasive monitoring of oxidative stress biomarkers in human sweat (Figure 8B), specifically hydrogen peroxide (H_2_O_2_) and phosphorylated proteins [125]. Ying et al. constructed a CNTs/MoS_2_-X coaxial core-shell structure by modifying Prussian blue on the electrode and incorporating sulfur-deficient molybdenum disulfide (MoS_2_-X) onto multi-walled carbon nanotubes (CNTs). This structure was further synthesized with highly dispersed titanium dioxide NPs (TiO_2_) to form a CNTs/MoS_2_-X/TiO_2_ composite material. This structure not only increased the electrochemically active surface area, providing more reaction sites, but also modulated the electronic structure of the material through sulfur vacancies and defect engineering, thereby enhancing its electrochemical response to target molecules. Additionally, the introduction of TiO_2_ leverages its lipophilic ion-exchange properties, improving the enrichment capability for phosphorylated proteins, thereby significantly boosting the sensor’s electrochemical activity and sensitivity. This sensor can rapidly and sensitively detect H_2_O_2_ and phosphorylated proteins, featuring an exceptionally low detection threshold and a broad linear detection range. Moreover, the integrated microfluidic device effectively facilitates the gathering and conveyance of sweat, further enhancing detection efficiency. Biocompatibility assessments have confirmed the excellent biocompatibility and safety of the electrode materials. The practical application test results demonstrate that the sensor can effectively distinguish the concentration differences of H_2_O_2_ and phosphorylated proteins in the sweat of healthy volunteers and elderly individuals with neurodegenerative diseases, providing a powerful new tool for monitoring disease markers related to oxidative stress.

Inadequate sweat volume can lead to a low concentration of analytes, thereby restricting the effectiveness of monitoring. To address this issue, Zhou et al. developed an autonomous sweating wearable platform for noninvasive monitoring of bilirubin levels [126]. Bilirubin is a biomarker associated with various physiological functions and health conditions, typically measured through blood tests. Abnormal bilirubin metabolism can lead to severe health issues, such as neonatal brain damage or even death. The platform incorporates an autonomous heating film that generates heat through the electrothermal effect, stimulating sweat glands on the skin surface to induce sweat secretion. The sweat sample is transported through a microfluidic system to flexible electrodes, that have been surface-modified with a composite material of MXene and Multi-Walled Carbon Nanotubes (MXene/MWCNT). The MXene/MWCNT composite exhibits a large specific surface area and excellent electrochemical activity, enabling efficient electrochemical detection of bilirubin while minimizing interference from ascorbic acid. The sensor has a detection range of 0.005 mg/dL to 0.6 mg/dL, making bilirubin monitoring more convenient. Furthermore, the platform can be redesigned to monitor other sweat biomarkers. Cui et al. developed an innovative skin-wearable nanobiosensor that utilizes gold NPs and MXene materials, achieving ultra-high sensitivity detection of estradiol in sweat through a target-induced strand displacement reaction, with a detection limit of 0.14 pM [127]. The study integrated a microfluidic system capable of automatically inducing sweat secretion and controlling sweat sampling with precise capillary bursting valves. Gold NPs and MXene, as key nanomaterials, have significantly enhanced the performance of wearable nanobiosensors. AuNPs improve the sensitivity and response speed of the sensors by increasing the electrode surface area and enhancing electron transport. MXene further enhances the selectivity and stability of the sensors by improving electrode conductivity and providing anti-interference properties. Additionally, the integrated sensor can monitor and calibrate the pH, ionic strength, and temperature of sweat in real time, ensuring detection accuracy. Human trials validated that the sensor accurately monitors estradiol levels in sweat, which are highly correlated with serum estradiol levels, exhibiting periodic fluctuations during the menstrual cycle. This technology not only provides a new, non-invasive tool for women’s health management and fertility monitoring but also demonstrates the significant potential of microfluidic technology in personalized medical monitoring.

Currently, the clinical assessment of C-reactive protein (CRP) relies on invasive blood sampling, and existing commercial point-of-care testing devices are bulky and unable to achieve picomolar-level sensitivity for CRP in sweat. Tu et al. designed a wearable wireless patch—InflaStat—capable of real-time electrochemical detection of the inflammatory biomarker CRP in sweat [128]. AuNPs enhance the sensitivity of CRP detection by increasing binding sites and facilitating electron transfer, while also serving as signal amplification carriers to boost detection signals. They synergize with a graphene substrate to enable simultaneous detection of multiple parameters. They achieved automated sampling, mixing, and detection of CRP in sweat by designing specific microfluidic pathways. With the ability to detect a minimum CRP concentration of 8 pM, this sensor presents the opportunity for early diagnosis and monitoring of a range of diseases.

In addition to extracting biomarkers through sweat, the analysis of interstitial fluid also serves as an effective approach. M. R. et al. developed a wearable microneedle sensor based on a biopolymer-protected graphene oxide (GO)-Fe_3_O_4_ nanocomposite for real-time and continuous monitoring of dopamine levels (Figure 8C) [129]. Abnormal dopamine levels are associated with various neurological disorders, including Segawa disease and AD. The sensor utilizes a chitosan-protected Fe_3_O_4_-GO composite as the chemical recognition element, coated with a Nafion anti-fouling layer, and detects dopamine through square wave voltammetry and chronoamperometry. GO exhibits excellent electrical conductivity, large specific surface area, high mechanical strength, rapid charge mobility, and abundant surface functional groups, making it an outstanding electrocatalyst and electron transport medium in electrochemical sensors, thereby significantly enhancing sensor sensitivity. In phosphate buffer and artificial interstitial fluid, the sensor demonstrated a low detection limit (90 nM to 0.6 mM) and excellent analytical performance. Furthermore, the microneedle sensor exhibited good long-term storage stability, reproducibility, and sensitivity in a gel model simulating skin, offering an innovative monitoring instrument for the diagnosis and treatment of neuropsychiatric disorders. Although the performance of the sensor has been validated in artificial interstitial fluid and simulated skin gel models, there is a lack of testing in real human environments. The complexity of human skin and interstitial fluid is significantly higher than that of artificial simulated environments, which may introduce more interfering substances that affect the actual performance of the sensor. Future research needs to focus on in vivo experiments and develop more efficient antipollution coatings.

### 4.2. Medical Imaging

Medical imaging is essential for effective diagnostics, and the application of nanomaterials in this area has achieved remarkable success. Nanomaterials have greatly improved the accuracy of imaging detection, developing notable advancements in this field [134,135]. Microfluidic technology can synthesize NPs with specific functions, which can be used as contrast agents for magnetic resonance imaging (MRI), significantly enhancing image quality. Kafali et al. synthesized chitosan-superparamagnetic iron oxide composite nanoparticles (Ch-SPIONs) using microfluidic technology, demonstrating significant potential in the field of medical imaging [130]. The r1 and r2 relaxivities are critical indicators for evaluating the efficacy of NPs as MRI contrast agents, with NPs exhibiting high r2 relaxivities producing stronger negative contrast effects in imaging. Ch-SPIONs possess high magnetization values, exhibit stronger responses in magnetic fields, and can more effectively influence the relaxation processes of surrounding protons, thereby enhancing relaxivities and improving MRI signals. Ch-SPIONs not only significantly enhance the contrast and clarity of MRI images but also enable precise targeted imaging under the guidance of external magnetic fields. Additionally, the excellent biocompatibility of Ch-SPIONs with osteoblasts and their significant antibacterial activity against harmful bacteria provide a solid foundation for their broad application in the diagnosis, monitoring, and treatment of infectious diseases. Nevertheless, further in-depth in vivo studies are still required to ensure the safety and efficacy of Ch-SPIONs in practical clinical applications.

Microfluidic technology also enables the rapid and continuous preparation of high-density lipoprotein (HDL) nanobiologics containing surface and core payloads (hydrophobic drugs, nanocrystals, or polymers) [131]. Mulder et al. utilized microfluidic technology to incorporate various imaging labels into HDL nanobiologics, such as chelated radionuclides or paramagnetic and fluorescent lipids, making them suitable for multiple imaging techniques, including positron emission tomography, magnetic resonance imaging, and computed tomography. These functionalized HDL nanobiologics not only facilitate tracking and molecular imaging but also serve as diagnostically active contrast agents, offering new perspectives for in vivo studies of cardiovascular diseases and cancer models. The application of microfluidic technology significantly enhances the potential and utility of HDL nanobiologics in precision medicine and imaging diagnostics.

## 5. Conclusions and Perspectives

The integration of nanomaterials and microfluidic devices technology has significantly advanced the progress of disease diagnosis and treatment, leveraging the benefits of nanomaterials such as high surface-to-volume ratio, inherent surface plasmon effects or magnetic resonance properties, easy surface chemical modification, and the precise control offered by microfluidic technology along with minimal sample requirements for disease diagnosis. The occurrence of diseases such as tumors, neurological disorders, and cardiovascular diseases is closely associated with the presence of numerous biomarkers. By combining various types of microfluidic chips with nanomaterials and employing diverse technological approaches, rapid identification and detection of biomarkers have been achieved, making revolutionary contributions to early disease diagnosis [136,137,138]. At the same time, microfluidic devices evaluate and improve treatment methods by reconstructing the in vivo 3D model and microenvironment combined with nanomaterials. The therapeutic potential of a variety of nanomedicines has been successfully demonstrated in different disease models. The study of nanomedicines provides an innovative strategy for the treatment of these diseases. In the future, new medical technologies are poised to be pivotal for the advancement of the healthcare industry. The fusion of nanomaterials and microfluidic devices has demonstrated significant potential in this sector, spearheading innovation in medical technology [122,123]. This amalgamation of technologies not only drives advancements in the medical domain but also unlocks fresh opportunities for future medical solutions.

While the convergence of nanomaterials and microfluidic devices has made considerable strides in the realm of disease diagnosis and treatment, the practical clinical application of integrating microfluidic devices and nanomaterials encounters numerous challenges.

### 5.1. Feasibility and Clinical Transformation

While microfluidic organ chips construct disease models that closely mimic the human microenvironment, they remain singular models without the influence of other biological factors. Additionally, the detection accuracy of microfluidic technology for clinical samples requires further enhancement. The translation of laboratory technologies to clinical settings still presents challenges. Thus, future research necessitates more clinical trials to meet the demands of widespread commercial and medical applications. The rapid development of artificial intelligence (AI) currently provides a lot of opportunities to enhance microfluidic platforms, using its predictive capabilities, data analysis, and efficient data processing. AI is expected to catalyze the potential of microfluidics in establishing automated drug screening systems [139]. By significantly boosting detection efficiency and improving the accuracy and robustness of analyses through multiple detections, AI helps guide clinicians in making real-time clinical decisions [140].

Moreover, the application of this technology also faces regulatory obstacles. Firstly, there is the issue of classification ambiguity: microfluidic devices incorporating nanomaterials may be categorized as medical devices (such as IVD), drugs (such as nanomedicine), or combination products, resulting in unclear regulatory pathways. For instance, the U.S. FDA requires determining the leading regulatory authority based on the primary mechanism of action. Secondly, the lack of unified standards is a concern; there are currently no global uniform standards for the characterization of nanomaterials (such as particle size distribution and surface modification) and the performance of microfluidic devices (such as flow rate control and detection limits). The differences in regulatory requirements across different regions may hinder multi-center clinical trials.

### 5.2. Safety of Nanomaterials

#### 5.2.1. Storage and Preparation of Nanomaterials

Each nanomaterial has its unique scope of application and can exhibit optimal performance only under specific conditions. For example, the stability and sterilization of industrially produced lipid nanoparticles (LNPs) require special attention, as there are many factors that affect their stability. Inappropriate storage conditions and excessive storage time may affect the stability of LNPs, which may in turn cause toxic side effects and affect the final therapeutic effect [141]. These problems also exist in the preparation of other nanomaterials. Therefore, it is necessary to find appropriate strategies to ensure the absolute safety of nanomaterials during industrial preparation, and it is urgent to standardize nanotechnology to obtain convincing and reproducible results. Currently, existing research primarily focuses on optimizing the synthesis and processing of nanomaterials to enhance their stability, such as using ligands and altering the size of nanomaterials through doping with other ions [142]. Additionally, advanced materials engineering strategies are being developed to address issues encountered during the storage of nanomaterials, including lyophilization, ultrasonic treatment [143], and coating techniques [144]. Future efforts need to further improve production processes, explore innovative synthesis techniques to overcome cost and scalability challenges, and establish relevant guidelines to form standardized production processes and regulatory frameworks.

#### 5.2.2. Blood Compatibility of Nanomaterials

Nanomaterials can interact with blood cells after entering the bloodstream, potentially triggering a series of adverse reactions. Tkachenko indicates that nanomaterials may promote erythrocyte lysis, affect blood coagulation, alter phagocytosis, and upregulate proinflammatory cytokines [145]. To ensure the safety of nanomaterials in medical applications, it is essential to evaluate their blood compatibility. Rodrigues et al. utilized a microfluidic device to evaluate the effect of magnetic nanoparticles (MNPs) on the deformation index (DI) of red blood cells (RBCs) [146]. As the concentration of MNPs increased, the DI value of the RBCs decreased. However, hemolysis analysis indicated that MNPs exhibit good blood compatibility. Lage et al. also utilized microfluidic devices to discover that the decreasing trend of DI was associated with an increase in MNPs concentration [147]. Although MNPs influences RBC DI at certain concentrations, this impact is not significant, indicating a degree of biocompatibility for biomedical applications. Naskar et al. discovered that the addition of nanomaterials can reduce platelet activation and thrombus formation using a microfluidic device [148], demonstrating good blood compatibility. However, Oddo et al. discovered using microfluidic chips that AgNPs can lower the half-maximal lethal concentration of leukocytes [149], indicating that dosage needs to be strictly controlled in practical applications.

Future research should further develop automated image analysis methods to enhance the assessment of NPs biocompatibility with speed and precision [150]. Additionally, it is crucial to conduct research on innovative surface modification materials. By incorporating specific chemical groups, biomolecules, or ligands onto the surfaces of nanomaterials, their stability and biocompatibility within biological systems can be significantly enhanced [151]. Concurrently, the development of green synthesis methods should be prioritized to mitigate the potential adverse effects of chemicals used in conventional synthesis processes on biological systems.

#### 5.2.3. Toxicity of Nanomaterials

Assessing the toxicity of nanomaterials helps to identify potential risks, thereby preventing diseases caused by improper use. The liver is a key organ for detoxification and drug metabolism, and NPs can cause varying degrees of hepatocellular toxicity. Microfluidic devices offer a high-throughput and physiologically relevant approach to screening and evaluating the safety of nanomaterials. Liu et al. discovered that AgNPs can induce hepatocyte damage and affect normal liver function at certain concentrations using microfluidic chips [152]. B. Esch et al. discovered that carboxylated polystyrene NPs are capable of penetrating intestinal epithelial cells and entering liver tissues, causing sublethal damage to hepatocytes at high doses [153]. The kidneys, as one of the excretory organs for NPs, are susceptible to damage caused by these nanomaterials. He et al. found that black phosphorus quantum dots accumulate in the kidneys and induce toxicity, with the mechanism involving endoplasmic reticulum stress mediated by the IRE1α signaling pathway [154]. Compared to traditional methods, toxicity assessments based on microfluidic systems facilitate understanding of potential mechanisms of action, guiding future design optimization. Despite the inherent toxicity of nanomaterials, modifying the surface of NPs to enhance their specificity can help reduce non-specific accumulation in the liver and kidneys, thereby alleviating toxicity [155].

In terms of the mechanisms of action of NPs, Dong et al. found that TiO_2_ NPs inhibited supporting cells and proliferating cells using a 3D microfluidic chip, leading to defects in spermatogenesis [156]. Nanomaterials can also induce mutations in specific genes and interfere with the electron transfer in mitochondria, leading to mitochondrial dysfunction [157]. NPs with a diameter of 20–30 nm can significantly disrupt the endothelial cell barrier function, leading to an increased permeability and loosening of the connections between endothelial cells. They enter the cells through caveolae/raft-mediated endocytosis [158]. Therefore, precise modulation of these signaling pathways can effectively counteract the potential cytotoxicity and other adverse effects of NPs, such as using specific calcium channel blockers to significantly alleviate the impact of nanomaterials on the endothelial barrier function. Size and shape are the primary factors influencing NPs’ entry into cells. Strict control of NPs’ size and shape during synthesis is expected to reduce toxicity and oxidative stress. Additionally, certain surface modifications and charge treatments can enhance nanoparticle application feasibility [159].

### 5.3. Nanoparticle Production

Reproducible synthesis of batches of NPs remains difficult. The performance of nanomaterials is heavily influenced by their quality, including factors such as size, shape, and uniformity. As a result, achieving consistent properties across batches of NPs in quantities suitable for clinical applications remains problematic. One potential solution to this issue lies in high-throughput and parallel microfluidic synthesis techniques, which have the capability to address these challenges. Currently, microfluidic synthesis has utilized the design of more parallel channels to further enhance the yield of nanomaterials [160]. Microfluidics significantly improves the reproducibility and controllability of NPs synthesis by precisely controlling the synthesis parameters and mixing intensity [161]. This provides a promising method for the large-scale commercial production of nanomaterials. However, due to the instability of the manufacturing process, there may still be differences in the size and quality of the products between batches. Therefore, further optimization of the process is required to ensure the reproducibility of nanomaterial production. AI-driven automated synthesis platforms can not only quickly identify optimal reaction conditions, but also quickly recover to the optimal state when encountering interference, thereby ensuring the stability of the nanomaterial synthesis process and the high quality of the product [162]. Establishing an automated platform suitable for the synthesis of various nanomaterials will greatly improve the yield of nanomaterials and reduce costs.

### 5.4. Industrial Standardization of Microfluidic Technology

Ensuring the consistency of industrial production environments is crucial. Replicating microfluidic chips from laboratory to industrial settings presents challenges due to inherent differences and limitations between these environments. In addition, most current microfluidic devices are custom designed and manufactured for certain specific applications and cannot meet the requirements of large-scale commercial applications. The wider implementation of these systems will require the clinical community to use more standardized microfluidic systems. By overcoming these challenges, we believe that nanomaterial-incorporated microfluidic devices will be widely used in disease diagnosis and treatment research and will become a powerful tool for future basic research and clinical applications.

### 5.5. Novel Detection Strategies

Relying solely on a single biomarker for clinical decision-making may lack reliability, highlighting the potential value in developing microfluidic systems capable of simultaneous analysis of diverse biomarkers. Additionally, there is a longstanding anticipation for the advancement of microfluidic systems employing novel detection strategies, such as dual-mode detection utilizing specific nanomaterials, to unlock their robust application potential across varied contexts and environments. Moreover, the increasing demand for point-of-care testing underscores the significant relevance of disease detection through the use of nanomaterials and microfluidic technologies.

While numerous biomarkers have been successfully detected through the synergistic use of nanomaterials and microfluidic devices, further research is essential to broaden the scope of their applications. Simultaneously, it is expected that new nanomaterials and nanomedicines will be developed with the help of microfluidic devices, and multiple detection and drug screening of disease biomarkers can be achieved through microfluidic chips streamlining the detection process, reducing detection times, and facilitating the development of enhanced treatment strategies.

## Figures and Tables

**Table 1:** Summary of nanomaterials in disease detection applications.

Nanomaterial Name	Size	Shape	Function	References
gold nanobipyramids	85 ± 2 nm in length, 26 ± 4 nm in width	bicone	converting light energy into thermal energy, promoting the detection of cTnI	[32]
gold NPs	150 nm	sphere	binding with antibodies, enhancing the detection signal of hs-cTnT	[42]
CSWCNs, silicon NPs	N/D	tubular, sphere	constructing PHC barcodes	[43]
AIENPs, magnetic NPs	300 nm, N/D	sphere	as fluorescent probes and capture particles, detecting h-FABP	[44]
Ru(bpy)_3_^2+^ loaded silica NPs	250 nm	porous sphere	as ECL probes, enhancing the sensitivity of h-FABP detection	[45]
GNPs	40 nm	sphere	marking NT-proBNP immune complexes	[46]
SA-B-HRP	220 nm	sphere	enhancing the luminous signal, improving the detection sensitivity of PCT and IL-6	[47]
AgNPs, AuNPs	55.37 ± 6.7 nm, 40 ± 4.78 nm	sphere	detecting GFAP, enhancing signal	[48]
PS microspheres, AuNPs	5 μm, 40 ± 4.78 nm	sphere	enhancing signal	[49]
nanoporous membrane, AuNPs	41 ± 2 nm, N/D	nanopore structure, sphere	detecting Aβ, enhancing signal	[50]
Streptavidin-QD655	N/D	sphere	detecting ApoE	[51]
AuNPs	12–14 nm	sphere	detecting CTCs	[52]
AuNPs	5 nm	sphere	detecting CTCs	[53]
AuNCAs	N/D	tapered array	detecting ctDNA	[54]
SPM	1 μm	sphere	detecting ctDNA	[55]
P-mesh	N/D	network topology	detecting ctDNA	[56]
GNPs	15 nm	sphere	detecting HER2-positive exosomes	[57]
MIL-125-NH2 NPs	N/D	plate crystal	detecting CLD7	[58]
AuNCA	N/D	coronal structure	detecting hnRNPA1 and S100P	[59]
Fe_3_O_4_@AuNPs, AuNCs	N/D, 40 nm	sphere, hollow cube	enhancing signal	[60]
ZnMn_2_O_4_@rGO	N/D	sphere	improving the detection sensitivity of CEA	[61]

cTnI, cardiac troponin I; hs-cTnT, high-sensitivity cardiac troponin T; CSWCNs, carboxylated single-walled carbon nanotubes; N/D, not determined; PHC, photonic crystal; AIENPs, aggregation-induced emission nanoparticles; h-FABP, heart-type fatty acid-binding protein; ECL electrochemiluminescence; GNPs, gold nanoparticles; NT-proBNP, N-terminal pro B-type natriuretic peptide; SA-B-HRP, streptavidin-biotin-horseradish peroxidase; PCT, procalcitonin; IL-6, interleukin-6; AgNPs, silver nanoparticles; AuNPs, gold nanoparticles; GFAP, glial fibrillary acidic protein; PS, polystyrene; Aβ, amyloid β; Streptavidin-QD655, Streptavidin-quantum dots655; ApoE, Apolipoprotein E; CTCs, circulating tumor cells; AuNCAs, Au nanocone arrays; ctDNA, circulating tumor DNA; SPM, superparamagnetic; P-mesh, padlock probes-conjugated nanomesh; MIL-125-NH2, Materials Institute Lavoisier; CLD7, Claudin7; AuNCA, Au nanocrown array; hnRNPA1, heterogeneous nuclear ribonucleoprotein A1; S100P, S100 calcium-binding protein P; Fe_3_O_4_@AuNPs, gold-coated iron tetroxide particles; AuNCs, gold nanocages; ZnMn_2_O_4_@rGO, ZnMn_2_o_4_@reduced graphene oxide; CEA, carcinoembryonic antigen.

**Table 2:** Comparison of the advantages and disadvantages of different types of detection methods.

Type	Methods	Advantages	Disadvantages	References
Traditional	ELISA	simple operation, high sensitivity	false positive results, cross-reactivity, prolonged duration	[88]
Novel	optical detection (SERS, LSPR and others)	high sensitivity, high specificity, multiple detection	complex equipment, signal interference	[42,46,49,54,57,59,60]
	electrochemical detection	high sensitivity, fast response	limited selectivity, changes in the stability of the electrode	[45,50,51,52,58,61]
	hot plasma detection	high sensitivity, rapid response, multiple detection	high equipment cost, variation in thermal stability of nanomaterials	[32]
	fluorescence detection	high sensitivity, high specificity, multiple detection	prolonged photobleaching, background signal interference	[44,53,56]
	magnetic separation technology	efficient separation, simple operation	sample requires pretreatment, changes in the stability of magnetic beads	[55]
	chemiluminescence immune assay	high sensitivity, high specificity	poor stability of reagents and substrates, complicated operation	[47]

ELISA, enzyme-linked immunosorbent assays; SERS, surface-enhanced Raman spectroscopy; LSPR, localized surface plasmon resonance.

**Table 4:** Summary of nanomaterials in novel medical applications.

Nanomaterial Name	Size	Shape	Function	References
FC-ZnONRs, LIG	N/D	flower cluster structure, 3D porous structure	improving the electrochemical detection sensitivity of copper ions, increasing electrical conductivity	[124]
CNTs/MoS2-X/TiO_2_	N/D	core-shell structure	detecting H_2_O_2_ and phosphorylated proteins	[125]
MXene/MWCNT	N/D	core-shell structure	enhancing the signal of bilirubin	[126]
AuNPs, MXene	22 nm, N/D	sphere, stratified structure	enhancing the signal of estradiol	[127]
AuNPs	20 nm	sphere	detecting CRP	[128]
Fe_3_O_4_, GO	N/D	sphere, stratified structure	improving the electrochemical detection sensitivity of dopamine	[129]
Ch-SPIONs	8.8 ± 1.2 nm	sphere	as MRI contrast agent, enhancing image contrast and clarity	[130]
HDL nanobiologics	8–9 nm, 20–30 nm, 40–400 nm	disc, sphere	integrating multiple imaging labels, suiting various imaging technologies	[131]

FC-ZnONRs, flower cluster-shaped zinc oxide nanorods; LIG, laser-induced graphene; N/D, not determined; CNTs/MoS2-X/TiO_2_, Carbon Nanotubes/Molybdenum Disulfide with Sulfur Vacancies/Titanium Dioxide; H_2_O_2_, hydrogen peroxide; MXene/MWCNT, Mxene/Multi-Walled Carbon Nanotubes; AuNPs, gold nanoparticles; CRP, C-reactive protein; Fe_3_O_4_, Iron (III) oxide; GO, graphene oxide; Ch-SPIONs, chitosan-superparamagnetic iron oxide composite nanoparticles; MRI, magnetic resonance imaging; HDL, high-density lipoprotein.

## Data Availability

No primary research results, software, or code have been included and no new data were generated or analyzed as part of this review.

## Abbreviations

The following abbreviations are used in this manuscript:

NPs	Nanoparticles
EDL	Electrical Double Layer
CT	Computed tomography
CVD	Cardiovascular diseases
WHO	World Health Organization
cTnI	Cardiac troponin I
LSPR	Localized surface plasmon resonance
SERS	Surface-enhanced Raman spectroscopy
ELISA	Enzyme-linked immunosorbent assays
hs-cTnT	High-sensitivity cardiac troponin T
PhC	Photonic crystal
CSWCNs	Carboxylated single-walled carbon nanotubes
Myo	Myoglobin
BNP	B-type natriuretic peptide
h-FABP	Heart-type fatty acid-binding protein
AMI	Acute myocardial infarction
AIENPs	Aggregation-induced emission nanoparticles
ECL	Electrochemiluminescence
NT-proBNP	N-terminal pro B-type natriuretic peptide
POC	Point-of-care
GNPs	Gold nanoparticles
PCT	Procalcitonin
IL-6	Interleukin-6
SA-B-HRP	Streptavidin-biotin-horseradish peroxidase
MIS	Microfluidic immunoassay system
SA	Streptavidin
B	Biotin
HRP	Horseradish peroxidase
AD	Alzheimer’s disease
PD	Parkinson’s disease
MS	Multiple sclerosis
Aβ	Amyloid β
P-tau	Phosphorylated tau protein
GFAP	Glial fibrillary acidic protein
NanoPADs	Microfluidic device for SERS immunoassay based on nanocellulose paper
AgNPs	Silver nanoparticles
PS	Polystyrene
AuNPs	Gold nanoparticles
OECT	Organic electrochemical transistor
μf-OECT	Microfluidic integrated organic electrochemical transistor
ApoE	Apolipoprotein E
SPE	Screen-printed electrodes
Streptavidin-QD655	Streptavidin-quantum dots655
CTCs	Circulating tumor cells
ctDNA	Circulating tumor DNA
DEP	Dielectrophoresis
PDMS	Polydimethylsiloxane
AuNCAs	Au nanocone arrays
SPM	Superparamagnetic
P-mesh	Padlock probes-conjugated nanomesh
EVs	Extracellular vesicles
CLD7	Claudin7
CRC	Colorectal cancer
MIL-125-NH2	Materials Institute Lavoisier
hnRNPA1	Heterogeneous nuclear ribonucleoprotein A1
S100P	S100 calcium-binding protein P
AuNCA	Au nanocrown array
MMP-9	Matrix metalloproteinase-9
Fe_3_O_4_@AuNPs	Gold-coated iron tetroxide particles
AuNCs	Gold nanocages
CEA	Carcinoembryonic antigen
ZnMn_2_O_4_@rGO	ZnMn_2_o_4_@reduced graphene oxide
MFC	Microfluidic chip
RGNps	Resveratrol gold nanoparticles
CGNps	Citrate gold nanoparticles
HUVECs	Human umbilical vein endothelial cells
ROS	Reactive oxygen species
pNPs	Polystyrene nanoparticles
GCPIH	Glycol chitosan-polypyrrole-iron oxide-heparin
tPA	Tissue plasminogen activator
tPA-DPNs	Tpa-discoidal polymeric nanoconstructs
BBB	Blood–brain barrier
μBBB	Blood–brain barrier microfluidic model
Tf@pSiNPs	Transferrin-functionalized porous silicon nanoparticles
BSA@pSiNPs	Bovine Serum Albumin-functionalized porous silicon
GBM	Glioblastoma multiforme
CDDP	Cisplatin
TMZ	Temozolomide
PEG	Polyethylene glycol
POM	Polyoxometalates
D-T7/Tet1-lipids@PL	D-T7/Tet1-lipids@PLGA-Lamotrigine nanoparticles
LTG	Lamotrigine
U87-MG	Glioblastoma multiforme
DTXL	Docetaxel
DTXL-SPN	Docetaxel-spherical polymeric nanoparticles
HA	Hyaluronic acid
GEM	Gemcitabine
IMQ	Imiquimod
IMQ-HA-GEM	Hyaluronic acid-gemcitabine-imiquimod
GelMA	Gelatin methacryloyl
MUC1	Mucin1
HGNs	Hollow gold nanoshells
HGNs@anti-MUC1	PEG
PTT	Photothermal therapy
TVOC	Tumor vasculature-on-a-chip
ECM	Extracellular matrix
TNF-α	Tumor necrosis factor-α
PEG-PLGA NPs	Poly(ethylene glycol))/poly(lactide-co-glycolic acid) nanoparticles
FA	Folic acid
TMOC	Tumor microenvironment-on-a-Chip
PTX	Paclitaxel
PTX-PLGA-SH NPs	PTX-loaded nanoparticles
LIG	Laser-induced graphene
FC-ZnONRs	Flower cluster-shaped zinc oxide nanorods
H_2_O_2_	Hydrogen peroxide
MoS_2_-X	Molybdenum disulfide with sulfur vacancies
CNTs	Carbon nanotubes
TiO_2_	Titanium dioxide
CNTs/MoS_2_-X/TiO_2_	Carbon Nanotubes/Molybdenum Disulfide with Sulfur Vacancies/Titanium Dioxide
MXene/MWCNT	Mxene/Multi-Walled Carbon Nanotubes
CRP	C-reactive protein
GO	Graphene oxide
Fe_3_O_4_-GO	Iron (III) oxide-Graphene Oxide
MRI	Magnetic resonance imaging
Ch-SPIONs	Chitosan-superparamagnetic iron oxide composite nanoparticles
HDL	High-density lipoprotein
AI	Artificial intelligence
IVD	In vitro diagnostic products
U.S. FDA	U.S. Food and Drug Administration
LNPs	Lipid nanoparticles
MNPs	Magnetic nanoparticles
DI	Deformation index
RBCs	Red blood cells
